# Multi-responsive nanocomposite hydrogel for synergistic photothermal-chemotherapy to prevent postoperative recurrence and metastasis of uveal melanoma

**DOI:** 10.1016/j.mtbio.2025.102628

**Published:** 2025-12-03

**Authors:** Zhihao Guo, Jiangcheng Tan, Junjie Chen, Jieqiong Liu, Wei Xiao, Xinyuan Huang, Cailing Wei, Ruohua Zhu, Ji-Liang Li

**Affiliations:** a National Engineering Research Center of Ophthalmology and Optometry, School of Biomedical Engineering, Eye Hospital, Wenzhou Medical University, Wenzhou, 325027, China; b Wenzhou Institute, University of Chinese Academy of Sciences, Wenzhou, 325000, China; c Hangzhou GreenIce EcoTech Co., Ltd., Hangzhou, 311200, China

**Keywords:** Uveal melanoma, Nanocomposite hydrogel, Photothermal therapy, Drug delivery, Recurrence and metastasis

## Abstract

Postoperative recurrence and metastasis remain major challenges in improving the prognosis of uveal melanoma (UM). Herein, we developed a chitosan-based injectable multi-responsive nanocomposite hydrogel (CPT NPs gel) for localized and synergistic photothermal-chemotherapy following tumor resection. This hydrogel co-encapsulates pH/GSH-responsive camptothecin-loaded nanoparticles (CPT NPs) and polydopamine, forming a porous, self-healing network that persists in vivo for over 28 days. Upon near-infrared (NIR) irradiation, the hydrogel rapidly elevates the local temperature to approximately 60 °C within 40 s, enabling efficient ablation of residual tumor tissue at the surgical site. Concurrently, CPT NPs are sustainably released from the hydrogel and enter systemic circulation, where they undergo charge reversal upon encountering the mildly acidic microenvironment of metastatic lesions to enhance cellular uptake and subsequently disassemble in response to intracellular GSH, thereby eliminating disseminated UM cells. In a partially resected MuM-2B tumor-bearing mouse model, CPT NPs gel combined with NIR irradiation completely abrogated local tumor recurrence and significantly reduced pulmonary metastasis, limiting metastatic infiltration to less than 1 % without observable systemic toxicity. Overall, this study presents a promising multifunctional therapeutic platform for the synergistic prevention of UM recurrence and metastasis.

## Graphical abstract

**Figure:**
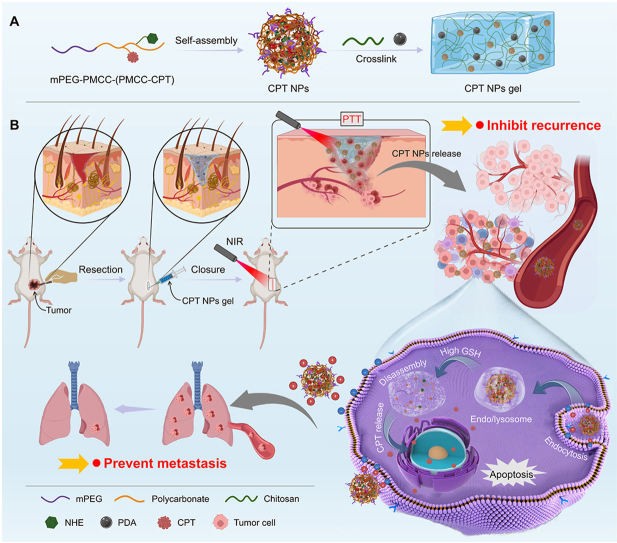

## 1 Introduction

Uveal melanoma (UM) is the most common primary intraocular malignancy in adults [1]. Surgical intervention remains the mainstay for controlling local tumor progression. However, microscopic residual tumor cells frequently persist postoperatively, leading to recurrence and distant metastasis, which ultimately result in poor prognosis [2,3]. To mitigate this issue, adjuvant therapies such as chemotherapy and radiotherapy are routinely employed [4]. Unfortunately, these therapies are often accompanied by severe side effects and require repeated administration, imposing substantial physical and psychological burdens on patients [5]. Therefore, the development of safe and effective adjuvant strategies capable of eradicating residual tumor cells to prevent postoperative recurrence and metastasis is essential for improving the prognosis of UM patients.

Photothermal therapy (PTT) has emerged as a promising cancer treatment modality [6]. By employing photothermal agents that absorb near-infrared (NIR) light and convert it into heat, PTT induces irreversible thermal damage to tumor cells, thereby triggering apoptotic pathways [7]. Implanting these agents at the surgical site using biocompatible carriers, such as hydrogels, sponges or wafers, enables precise temperature regulation under external NIR irradiation. This localized hyperthermia selectively ablates residual tumor tissues while minimizing damage to surrounding healthy tissues [[8], [9], [10]]. Additionally, such implantable systems possess advantages including minimal invasiveness, operational simplicity, and the potential for sustained therapy through repeated irradiation sessions [11]. Nevertheless, PTT as a monotherapy is limited by tumor thermotolerance and reduced efficacy against disseminated tumor cells, which compromise its capacity to prevent postoperative recurrence and metastasis [[12], [13], [14], [15]].

Nanoparticle (NP)-based drug delivery systems provide an attractive complement to PTT. NPs can enhance the solubility and stability of poorly water-soluble agents and preferentially accumulate in tumor tissues through surface functionalization or intrinsic physicochemical properties [[16], [17], [18], [19], [20]]. Incorporation of stimuli-responsiveness allows NPs to respond to modest environmental changes in the tumor microenvironment, such as low pH, elevated enzyme concentrations, high glutathione (GSH) levels, and reactive oxygen species (ROS), triggering structural or chemical alterations that induce controlled drug release [21]. The intelligent release mechanism improves therapeutic precision and efficacy, compensating for the insufficient anti-metastatic capacity of PTT alone. Moreover, hyperthermia has been reported to overcome multidrug resistance by downregulating drug-efflux proteins [22]. Therefore, integrating NP-based chemotherapy with PTT constitutes a promising postoperative synergistic strategy to enhance therapeutic outcomes in UM.

Hydrogels have demonstrated considerable potential as multifunctional therapeutic platforms owing to their excellent biocompatibility, high drug encapsulation efficiency, and capacity for sustained release [[23], [24], [25]]. In particular, hydrogels integrating PTT and chemotherapy have shown remarkable potential for cancer treatment. However, most existing hydrogel systems mainly focus on localized tumor ablation. Although the released drugs can effectively kill tumor cells at the primary site, their therapeutic efficacy against circulating tumor cells remains limited, rendering them inadequate for eliminating highly metastatic malignancies such as UM [26,27]. Embedding drug-loaded NPs into hydrogel matrices to form nanocomposite hydrogels not only retains the intrinsic advantages of hydrogels but also enhances drug stability, tumor-targeting capability, and spatiotemporal release control [[28], [29], [30], [31], [32]]. Injectable nanocomposite hydrogels can be precisely delivered to the surgical site via minimally invasive administration, and rapidly conform to irregular postoperative cavities, forming a stable three-dimensional network in situ that serves as a physical barrier to prevent tissue adhesion and prolong local drug retention [8,33,34]. They can release NPs into systemic circulation to track and suppress metastatic lesions while mediating localized photothermal ablation at the postoperative site, thereby achieving combined local-systemic therapeutic benefits. Since the released NPs circulate through the bloodstream to reach metastatic sites, they should possess excellent colloidal stability to prevent aggregation and vascular occlusion [35]. pH-responsive charge-reversal NPs are particularly appealing for this purpose, as they maintain a negatively charged surface during circulation to prolong blood retention and minimize nonspecific interactions, while switching to a positive charge in the acidic tumor microenvironment to enhance cellular uptake and facilitate intracellular drug release. Compared with other stimuli-responsive systems that are constrained by the complexity, adaptability, and dynamics of the target tissue [36], this pH-triggered strategy leverages lactic acid-induced extracellular acidification resulting from accelerated tumor metabolism, offering a more universal and predictable mechanism for targeted delivery. Therefore, constructing a composite hydrogel system integrating PTT with acid-triggered charge-reversal NP-mediated chemotherapy holds significant promise for preventing postoperative UM recurrence while effectively inhibiting metastasis.

Among various hydrogel-forming materials, chitosan (CS), a natural polysaccharide derived from chitin, has attracted extensive attention due to its excellent biocompatibility, biodegradability, tissue adhesiveness, and antibacterial activity [37]. Its amino-functionalized backbone facilitates the formation of self-healing networks through dynamic covalent or physical interactions, highlighting CS as a promising candidate for the development of postoperative nanocomposite hydrogel systems [38]. Importantly, the acidic tumor microenvironment confers distinct advantages to Schiff base bonds formed between chitosan amino groups and aldehyde- or ketone-containing compounds. These dynamic bonds inherently provide both pH and shear responsiveness, enabling facile injection and controlled, efficient drug release specifically within the tumor microenvironment. Although various smart CS-based hydrogels have been developed, conventional small-molecule crosslinkers, such as formaldehyde or glutaraldehyde, are often associated with potential toxicity and limited biocompatibility [39,40]. Therefore, selecting appropriate crosslinkers to minimize the toxicity of multifunctional hydrogels remains crucial.

**Scheme 1:**
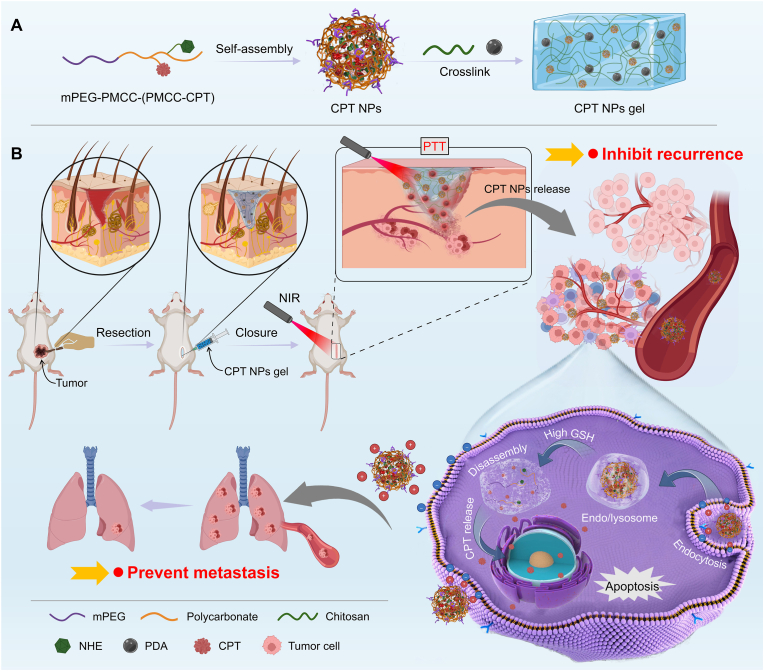
Schematic illustration of the fabrication of CPT NPs gel (A) and its therapeutic strategy for preventing postoperative recurrence and metastasis of UM (B).

Herein, we developed a multifunctional nanocomposite hydrogel (CPT NPs gel) by co-encapsulating the photothermal agent polydopamine (PDA) and stimuli-responsive camptothecin (CPT)-loaded nanoparticles (CPT NPs) within a CS-based matrix, aiming to synergistically prevent postoperative recurrence and metastasis of UM through combined photothermal therapy and chemotherapy (Scheme 1). In this system, PDA-mediated photothermal ablation serves as the first-line treatment, followed by gradual hydrogel degradation and sustained release of CPT NPs to eradicate residual tumor cells, thereby forming a spatiotemporally complementary and synergistic therapeutic platform for complete tumor elimination. CPT NPs were fabricated via self-assembly of a pH/GSH dual-responsive CPT prodrug polymer, mPEG-PMCC-(PMCC-CPT), which was synthesized by conjugating a disulfide-bridged CPT derivative and N-(2-hydroxyethyl)hexamethylenediamine onto a polycarbonate backbone. The NPs were engineered to undergo pH-responsive charge reversal from negative to positive in the mildly acidic tumor microenvironment for enhancing cellular uptake and drug utilization. The hydrogel network was formed through dynamic Schiff base

crosslinking between benzaldehyde-functionalized polyethylene glycol (PEG-CBA) and the amino groups of CS. The hydrophilic PEG-CBA functioned as a macromolecular crosslinker that not only ensured injectability and acid sensitivity but also endowed the hydrogel with excellent biocompatibility. Upon local injection at the postoperative UM site, PDA mediates rapid photothermal ablation of residual tumor cells under NIR irradiation, while the hydrogel matrix gradually degrades to facilitate sustained release of CPT NPs. These NPs target metastatic cells, undergo charge reversal to enhance cellular uptake, and release CPT in response to elevated intracellular GSH levels, thereby effectively eradicating disseminated tumor cells. The localized delivery strategy minimizes systemic toxicity, ensuring excellent biocompatibility and safety. Therefore, this multifunctional stimuli-responsive nanocomposite hydrogel provides a promising and translatable platform for synergistic prevention of UM recurrence and metastasis.

## 2 Materials and methods

### 2.1 Synthesis of mPEG-PMCC-(PMCC-CPT)

Synthesis of CPT-SS-OH: Camptothecin (0.79 g, 2.0 mmol) and 4-dimethylaminopyridine (DMAP, 0.78 g, 6.39 mmol) were dissolved in 50 mL of anhydrous dichloromethane (DCM) in a 100 mL round-bottom flask. Triphosgene (0.22 g, 0.74 mmol) was added under a nitrogen atmosphere, and the mixture was stirred for 30 min until it became clear. A solution of 2,2′-dithiodiethanol (1.54 g, 9.8 mmol) in 10 mL of anhydrous tetrahydrofuran (THF) was then added dropwise, and the reaction was allowed to proceed at room temperature for 24 h. Upon completion, the solvent was removed under reduced pressure. The residue was redissolved in 50 mL of DCM, washed sequentially with 50 mL of 1 N HCl and 50 mL of saturated brine, and dried over anhydrous sodium sulfate for 4 h. After filtration, the solvent was evaporated under reduced pressure to yield a pale yellow crude solid. Purification was carried out by column chromatography on silica gel (300–450 m^2^/g, 50–75 μm) using a glass column (32 mm diameter × 305 mm length) with ethyl acetate/methanol (10:1, v/v) as the eluent at an approximate flow rate of 4 mL/min, affording CPT-SS-OH as a yellow solid, which was further dried to constant weight under vacuum at 25 °C. ^1^H NMR (600 MHz, CDCl_3_) *δ* (ppm): 8.4 (s, 1H), 8.23 (d, 1H), 7.9 (d, 1H), 7.80 (m, 1H), 7.63 (m, 1H), 7.48 (s, 1H), 5.65 (m, 1H), 5.33–5.21 (m, 3H), 4.32 (m, 2H), 3.84 (m, 2H), 2.94–2.78 (m, 4H), 2.26–2.09 (m, 2H), 0.97 (m, 3H).

Synthesis of mPEG-*b*-PMBC: mPEG_113_ (4.0 g, 0.8 mmol), 5-methyl-5-benzyloxycarbonyl-1,3-dioxan-2-one (MBC, 12.0 g, 48 mmol), and DBU (0.12 mL, 0.8 mmol) were dissolved in anhydrous DCM (50 mL) under nitrogen and stirred at 25 °C for 24 h. The polymerization was quenched by adding benzoic acid (0.15 g, 1.2 mmol) in DCM. The solution was concentrated under reduced pressure and then slowly added dropwise into cold methanol using a Pasteur pipette at a rate of approximately one drop every 2 s to precipitate the polymer. The resulting colorless, transparent block copolymer (mPEG-*b*-PMBC) was collected and dried under vacuum at 25 °C for 24 h. ^1^H NMR (600 MHz, CDCl_3_) *δ* (ppm): 7.31 (m, Ar-***H***, 265H), 5.16 (s, Ar–C***H***_2_O–, 106H), 4.28 (s, C***H***_2_ in polycarbonate chain, 212H), 3.66 (s, C***H***_2_ in PEG, 452H), 1.23 (m, C***H***_3_ in polycarbonate chain, 159H).

Synthesis of mPEG-*b*-PMCC: mPEG-*b*-PMBC (10 g) was dissolved in a methanol/THF mixture (1:1, v/v, 300 mL), followed by addition of Pd/C (0.5 g) and Pd(OH)_2_/C (0.5 g) in a 500 mL round-bottom flask. After evacuation, the system was purged with hydrogen and stirred at room temperature for 48 h. The catalyst was removed by vacuum filtration, and the filtrate was concentrated under reduced pressure. The resulting product (mPEG-*b*-PMCC) was dried under vacuum at 25 °C to constant weight. ^1^H NMR (600 MHz, DMSO-*d*_6_) *δ* (ppm): 4.17 (m, C***H***_2_ in polycarbonate chain, 212H), 3.48 (s, C***H***_2_ in PEG, 452H), 1.13 (s, C***H***_3_ in polycarbonate chain, 159H).

Synthesis of mPEG-PMCC-(PMCC-CPT): mPEG-*b*-PMCC (0.51 g, 0.037 mmol) was dissolved in THF (30 mL). A solution of EDCI (0.192 g, 1 mmol) and DMAP (0.166 g, 1 mmol) in anhydrous DCM (10 mL) was added dropwise under stirring at room temperature. After 1 h of pre-activation, a DCM solution containing NHE (0.143 g, 1 mmol) and CPT-SS-OH (0.528 g, 1 mmol) was added slowly, and the reaction proceeded at room temperature for 48 h. The mixture was concentrated under reduced pressure, and the residue was added dropwise into cold methanol using a Pasteur pipette at a rate of approximately one drop every 2 s to precipitate the product. The resulting yellow solid was collected and designated as mPEG-PMCC-(PMCC-CPT). ^1^H NMR (600 MHz, DMSO-*d*_6_) *δ* (ppm): 8.71 (m, 22H), 8.18 (d, 44H), 7.91 (d, 22H), 7.76 (m, 22H), 7.11 (m, 22H), 5.59 (m, 44H), 5.32 (m, 44H), 4.4–3.7 (m, 336H), 3.57 (s, 452H), 3.05–2.8 (m, 88H), 1.28–0.98 (m, 369H).

### 2.2 Preparation of CPT-loaded NPs

Preparation of NPs: CPT-loaded NPs were prepared via the solvent exchange method. Briefly, mPEG-PMCC-(PMCC-CPT) (1 mg/mL) dissolved in 1,4-dioxane was placed in a dialysis bag (MWCO 3500 Da) and dialyzed against deionized water at room temperature for 72 h, with the external medium refreshed every 12 h. The resulting solution was filtered through a 0.45 μm membrane to obtain CPT NPs. Control NPs (Ctrl NPs) were prepared under the same conditions using polymer lacking the NHE group. All NP solutions were stored at 4 °C for further use.

### 2.3 Preparation of nanocomposite hydrogel

Synthesis of polydopamine (PDA): PDA was synthesized through the oxidative self-polymerization of dopamine hydrochloride under alkaline conditions. Briefly, dopamine hydrochloride (300 mg) was dissolved in deionized water (6 mL) and added to a mixed solution containing concentrated ammonia (0.6 mL), deionized water (60 mL), and ethanol (24 mL). The mixture was stirred for 48 h, after which PDA was collected by centrifugation (15,000 rpm), washed five times with deionized water, and finally freeze-dried to yield a black powder.

Synthesis of functionalized PEG (PEG-CBA): 4-Formylbenzoic acid (0.49 g, 3.26 mmol), DCC (0.84 g, 4.07 mmol), and DMAP (0.084 g) were dissolved in THF (25 mL) and stirred at room temperature for 0.5 h. PEG (3.26 g, 1.63 mmol) in THF (25 mL) was then added, and the reaction was allowed to proceed at room temperature for 48 h. The reaction was quenched with deionized water (1 mL), followed by filtration under reduced pressure through a 5.5 cm diameter filter paper with a pore size of 7–8 μm. The filtrate was dialyzed (MWCO 1000 Da) against water for 48 h, filtered through a 0.45 μm membrane, and lyophilized to yield PEG-CBA as a white solid.

Preparation of hydrogel: CS (300 mg), PDA (25 mg), and CPT NPs were dissolved in 1 mL of 2 % aqueous acetic acid (final CPT concentration: 1 mg/mL). Subsequently, PEG-CBA solution (50 μL, 33 wt%) was added and the mixture was vortexed at 2000 rpm for ∼30 s to form the CPT NPs gel. Other hydrogels were prepared in a similar manner: the CS gel consisted of CS and PEG-CBA; the PDA gel consisted of CS, PDA, and PEG-CBA; the free CPT gel consisted of CS, free CPT, and PEG-CBA. The gel microstructure was characterized by scanning electron microscopy (SEM, ZEISS Sigma 300).

### 2.4 Cell culture and animals

MuM-2B human invasive choroidal melanoma cells were obtained from the American Type Culture Collection and maintained in RPMI-1640 medium supplemented with 10 % fetal bovine serum and 1 % penicillin–streptomycin at 37 °C in a humidified incubator with 5 % CO_2_. Female BALB/c nude mice (5–6 weeks old) were purchased from Zhejiang Vital River Laboratories Co., Ltd (Zhejiang, China) and housed under standard conditions in sterile cages (five mice per cage). All animal procedures were conducted in accordance with the guidelines of the Experimental Animal Committee of Wenzhou Medical University. See additional details in supporting information.

### 2.5 Statistical analysis

Each experiment was performed in triplicate or with more specimens. Data are presented as mean ± standard deviation. Statistical analysis was carried out using Student's *t*-test for two groups' comparison or ANOVA for more than two groups' comparison. Statistical significance was denoted by asterisk(s) for ∗*P* < 0.05, ∗∗*P* < 0.01, ∗∗∗*P* < 0.001.

## 3 Results and discussion

### 3.1 Synthesis and characterization of mPEG-PMCC-(PMCC-CPT)

**Fig. 1:**
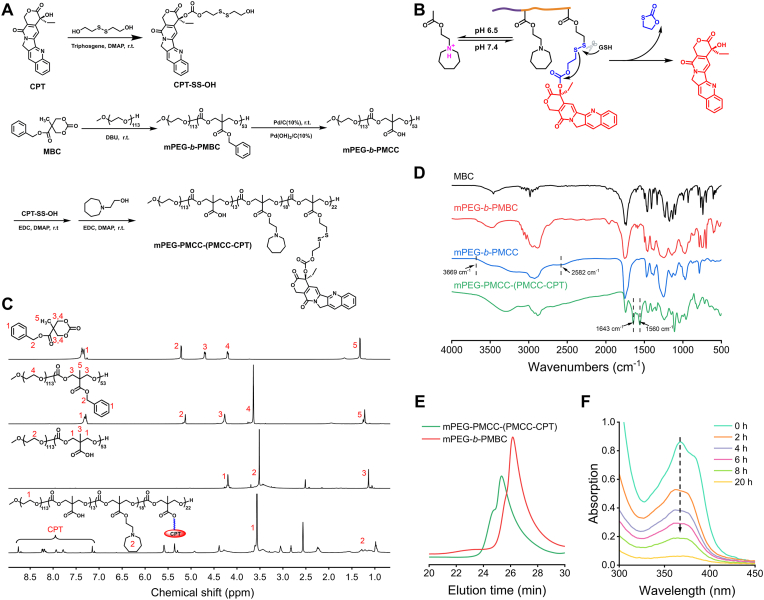
**Synthesis and characterization of mPEG-PMCC-(PMCC-CPT).** (A) Synthetic route of mPEG-PMCC-(PMCC-CPT). (B) Proposed pH/GSH-responsive mechanism of mPEG-PMCC-(PMCC-CPT). ^1^H NMR spectra (C), FT-IR spectra (D), and GPC profiles (E) of the synthesized polymers. (F) UV–vis absorption spectra of the 2 mg/mL mPEG-PMCC-(PMCC-CPT) solution containing 10 mM GSH.

The copolymer mPEG-PMCC-(PMCC-CPT) was synthesized through a multi-step process involving carbonate ring-opening polymerization, catalytic hydrogenation, and esterification (Fig. 1A). The resulting polymer was designed to exhibit pH/GSH dual-responsiveness: protonation of tertiary amines under mildly acidic conditions and disulfide bond cleavage in GSH-abundant environments, enabling controlled CPT release (Fig. 1B). The product structures were confirmed by proton nuclear magnetic resonance (^1^H NMR), Fourier-transform infrared spectroscopy (FT-IR), and gel permeation chromatography (GPC). The intermediate CPT-SS-OH was synthesized by conjugating CPT and bis(2-hydroxyethyl) disulfide, as verified by ^1^H NMR (Fig. S1) and FT-IR (Fig. S2). Compared with CPT, CPT-SS-OH displayed new ^1^H NMR signals at 4.32, 3.83, and 2.86 ppm, corresponding to methylene protons of the disulfide linker. Enhanced methylene C–H stretching bands at 2998–2778 cm^−1^ in the FT-IR spectrum further confirmed the successful conjugation. The block copolymer mPEG-*b*-PMBC was synthesized via mPEG-initiated ring-opening polymerization of MBC. The average degree of polycarbonate segment was calculated as 53 based on the integral ratio of peak 4.28 ppm–3.65 ppm in the ^1^H NMR spectrum (Fig. 1C). Benzyl protecting groups were subsequently removed via hydrogenolysis to obtain carboxyl-functionalized mPEG-*b*-PMCC. The disappearance of aromatic signals in the ^1^H NMR spectrum and the appearance of broad carboxyl stretching bands in the 3669–2582 cm^−1^ in the FT-IR spectrum (Fig. 1D) confirmed complete deprotection. The dual-responsive copolymer mPEG-PMCC-(PMCC-CPT) was synthesized by grafting CPT-SS-OH and N-(2-Hydroxyethyl)hexamethyleneimine (NHE) onto the side chains of mPEG-*b*-PMCC via esterification. In the ^1^H NMR spectrum, characteristic CPT peaks were observed at 8.73–7.11 ppm, while methylene proton signals at 1.28–1.18 ppm were attributed to NHE. The FT-IR spectrum further exhibited aromatic C=C stretching vibrations at 1643 and 1560 cm^−1^. The average grafting degrees of the CPT-SS-OH and NHE segments were calculated to be 22 and 18, respectively. GPC analysis (Fig. 1E) determined dispersity indices of 1.21 for mPEG-*b*-PMBC and 1.32 for mPEG-PMCC-(PMCC-CPT).

To assess the GSH sensitivity, mPEG-PMCC-(PMCC-CPT) was incubated with 10 mM GSH, and the release of CPT was monitored using a dialysis method. The characteristic UV absorption peak of CPT gradually decreased over time and became nearly undetectable after 20 h of incubation (Fig. 1F), indicating efficient GSH-triggered degradation of the copolymer.

### 3.2 Preparation and characterization of CPT NPs

**Fig. 2:**
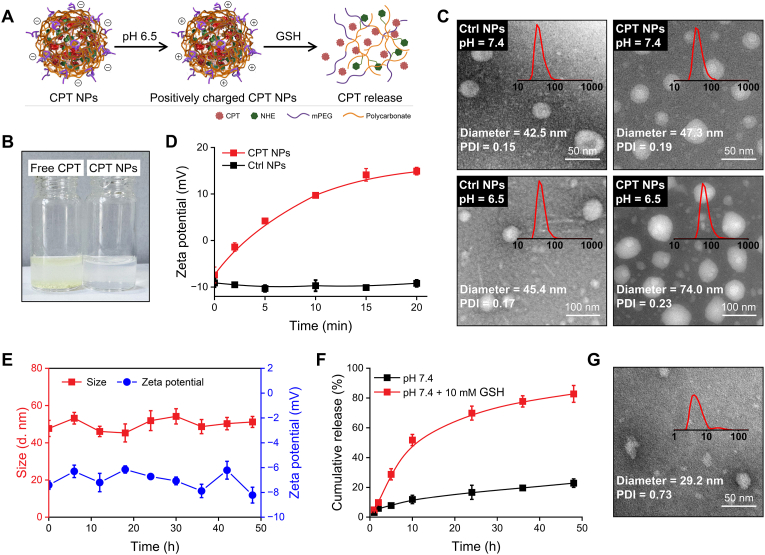
**Preparation and characterization of CPT NPs.** (A) Schematic of preparation and drug release of CPT NPs. (B) Comparison of water solubility between free CPT and CPT NPs. (C) TEM images and size distributions of Ctrl NPs and CPT NPs under different pH conditions. (D) Zeta potential changes of Ctrl NPs and CPT NPs at pH 6.5. (E) Size and zeta potential of CPT NPs after incubation in PBS containing 10 % FBS. (F) Cumulative release profiles of CPT from CPT NPs in PBS with or without 10 mM GSH. (G) TEM image and size distribution of CPT NPs after 48 h incubation in 10 mM GSH.

CPT NPs were prepared via the self-assembly of mPEG-PMCC-(PMCC-CPT), where the hydrophobic polycarbonate segments formed the inner core to encapsulate CPT, while the mPEG chains constituted a hydrophilic shell for stabilization and prolonged circulation. These NPs exhibited pH-responsive surface charge reversal, switching from negative to positive under mildly acidic conditions, and enabled rapid drug release in response to elevated intracellular GSH (Fig. 2A). Due to poor water solubility, conventional chemotherapeutics often suffer from limited bioavailability. Compared to free CPT, which precipitated in PBS, CPT NPs formed a uniform and transparent dispersion, indicating improved solubility (Fig. 2B), with a drug loading content of 22.4 %. To evaluate the charge-reversal behavior, control nanoparticles (Ctrl NPs) without NHE groups were prepared. TEM images revealed both Ctrl NPs and CPT NPs exhibited uniform spherical morphology, with hydrodynamic diameters of 42.5 nm and 47.3 nm at pH 7.4, respectively (Fig. 2C). Under mildly acidic conditions (pH 6.5), CPT NPs swelled to 74 nm, likely due to the protonation of tertiary amines [41], whereas Ctrl NPs remained essentially unchanged (45.4 nm). Consistently, CPT NPs underwent a rapid surface charge reversal from −7.4 mV to +4.2 mV within 5 min, reaching +14.9 mV at 20 min, while Ctrl NPs maintained a negative zeta potential (Fig. 2D). The colloidal stability of CPT NPs was further validated in PBS containing 10 % serum (Fig. 2E). After 48 h of incubation, no significant changes in particle size or zeta potential were observed. The dispersion remained transparent, without visible aggregation or sedimentation, suggesting effective resistance to nonspecific protein adsorption and favorable potential for prolonged systemic circulation. Additionally, CPT NPs exhibited an initially positive surface charge that progressively decreased in 10 % FBS-containing PBS at pH 6.5, reaching −1.8 mV after 12 h (Fig. S3). This reduction can be attributed to the electrostatic adsorption of negatively charged serum proteins onto the NP surface.

An effective antitumor drug delivery system should maintain systemic stability to prevent premature drug leakage while enabling rapid and stimulus-responsive drug release in the tumor-specific microenvironment [42]. To investigate the GSH-responsive drug release behavior of CPT NPs, in vitro release studies were performed using dialysis in PBS (pH 7.4) with or without 10 mM GSH (Fig. 2F). In the absence of GSH, only 22.8 % of CPT was released over 48 h, whereas GSH exposure markedly accelerated the process, resulting in 51.8 % release within 10 h and up to 82.8 % by 48 h. Post-release characterization revealed collapsed and irregular NP structures with an average diameter of 29.2 nm (PDI = 0.73), which was substantially smaller compared with the initial size (Fig. 2G). This distinctive release behavior can be ascribed to the cleavage of GSH-sensitive disulfide bonds in mPEG-PMCC-(PMCC-CPT), which triggers the dissociation of CPT from the polymer side chains, leading to the shrinkage of hydrophobic segments and ultimately destabilizing the NPs, thereby accelerating CPT release.

### 3.3 In vitro anti-tumor effects of CPT NPs

**Fig. 3:**
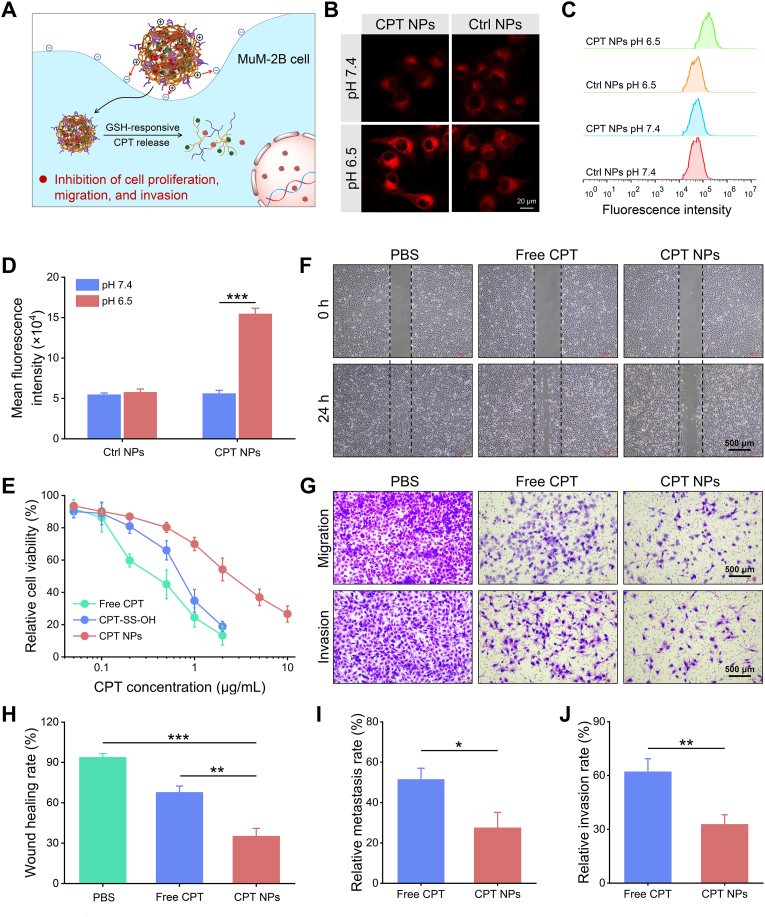
**In vitro anti-tumor effects of CPT NPs.** (A) Schematic illustration of CPT NPs-mediated in vitro anti-tumor effects. (B) CLSM images of MuM-2B cells incubated with CPT NPs or Ctrl NPs for 6 h; red fluorescence indicates intracellular CPT. (C) Flow cytometry analysis and (D) quantification of mean fluorescence intensity of MuM-2B cells treated with NPs under different pH conditions. (E) Relative cell viability of MuM-2B cells after 24 h incubation with various drug formulations. (F) Representative wound healing images of MuM-2B cells. (G) Transwell migration and invasion images of MuM-2B cells after 24 h. Quantitation of the wound closure (H), migration (I), and invasion assays (J) of MuM-2B cells.

Positively charged NPs typically exhibit enhanced cellular internalization due to electrostatic interactions with negatively charged moieties on the cell membrane [43]. Taking advantage of the pH/GSH dual-responsiveness, CPT NPs were rationally designed to undergo surface charge reversal within the acidic extracellular tumor microenvironment, thereby promoting cellular uptake. Once internalized, elevated intracellular GSH levels triggered rapid CPT release, ultimately enhancing antitumor efficacy (Fig. 3A).

To evaluate acid-enhanced cellular uptake, MuM-2B cells were incubated with CPT NPs or Ctrl NPs at pH 7.4 or 6.5 for 6 h. Intracellular fluorescence intensity was subsequently quantified using confocal laser scanning microscopy (CLSM) and flow cytometry. Both NP formulations produced detectable CPT fluorescence signals in MuM-2B cells (Fig. 3B). For Ctrl NPs, intracellular fluorescence intensities were comparable under the two pH conditions. In contrast, CPT NPs elicited markedly stronger fluorescence at pH 6.5 than at pH 7.4, and significantly higher than that of Ctrl NPs under the same acidic condition (Fig. S4). Flow cytometry further confirmed this pH-triggered enhancement in cellular uptake of CPT NPs (Fig. 3C). Notably, CPT NPs at pH 6.5 exhibited the highest mean intracellular fluorescence intensity, approximately 2.7-fold greater than that at pH 7.4 (Fig. 3D). These results indicate that acidity-induced charge reversal facilitates the cellular internalization of CPT NPs in MuM-2B cells.

The cytotoxicity of free CPT, CPT-SS-OH, and CPT NPs against MuM-2B cells was assessed using the CCK-8 assay (Fig. 3E). All formulations exhibited concentration-dependent cytotoxicity. The polymeric backbone mPEG-*b*-PMCC demonstrated excellent biocompatibility, maintaining over 80 % cell viability even at concentrations up to 200 μg/mL (Fig. S5), indicating that the cytotoxicity of CPT NPs was primarily attributable to the encapsulated CPT. Free CPT, which can rapidly diffuse into the nucleus and suppress cell proliferation, showed the strongest cytotoxicity with an IC_50_ of 0.37 ± 0.10 μg/mL (Fig. S6). By contrast, CPT NPs exhibited attenuated cytotoxicity, with an IC_50_ of 2.5 ± 0.46 μg/mL, significantly higher than those of free CPT and CPT-SS-OH. This attenuation in cytotoxic potency results from the reliance on endocytic uptake and the subsequent delay in intracellular GSH-triggered release of CPT from the NP core to reach therapeutically effective concentrations [44,45].

Given the high metastatic potential of UM, the anti-metastatic activity of CPT NPs against MuM-2B cells was systematically assessed using wound healing and transwell assays. To minimize confounding cytotoxicity and specifically evaluate inhibition of cell migration, the CPT concentration was fixed at 0.1 μg/mL. As shown in Fig. 3F, PBS-treated cells exhibited robust migratory activity, with 93.5 % wound closure after 24 h. In contrast, free CPT and CPT NPs significantly impeded wound healing, reducing the wound healing rates to 67.5 % and 34.9 %, respectively (Fig. 3H), suggesting potent anti-migratory effects. Consistent results were obtained in the transwell migration assays (Fig. 3G), where the number of migrated cells was markedly reduced following drug treatment, with relative migration rates of 51.4 % for free CPT and 27.3 % for CPT NPs (Fig. 3I). The superior inhibitory effect of CPT NPs is likely attributable to NP-mediated cellular uptake, leading to enhanced intracellular drug accumulation. Similarly, invasion assays demonstrated a pronounced reduction in invasive capacity, with invasion rates of 61.8 % for free CPT and 32.5 % for CPT NPs (Fig. 3J). Collectively, these results indicate that CPT NPs effectively suppress MuM-2B cell migration and invasion, highlighting their potential for preventing UM metastasis.

### 3.4 Preparation and characterization of nanocomposite hydrogel

**Fig. 4:**
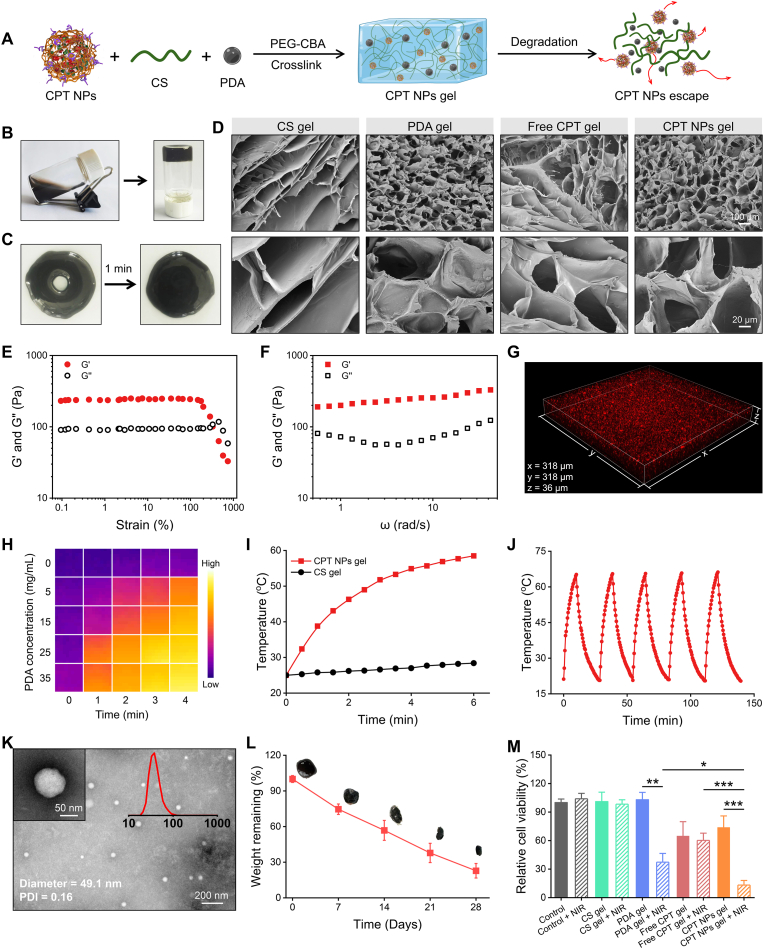
**Preparation and characterization of nanocomposite hydrogel.** (A) Schematic illustration of the preparation and drug release behavior of CPT NPs gel. (B) Photographs showing gelation of CPT NPs gel. (C) Self-healing behavior of CPT NPs gel. (D) Representative SEM images of different freeze-dried hydrogels. (E) Strain sweep and (F) frequency sweep of CPT NPs gel at 25 °C. (G) CLSM images of CPT NPs gel. (H) Infrared thermal images of hydrogel containing various concentrations of PDA under 808 nm laser irradiation (1 W/cm^2^). (I) Temperature elevation profiles of CPT NPs gel during 808 nm laser irradiation (1 W/cm^2^). (J) Cyclic photothermal performance of CPT NPs gel under repeated 808 nm laser irradiation (1 W/cm^2^). (K) TEM image and DLS analysis of NPs released from CPT NPs gel. (L) In vivo degradation profile of CPT NPs gel. (M) Relative viability of MuM-2B cells after 24 h incubation with different hydrogels.

The nanocomposite hydrogel (CPT NPs gel) was constructed by embedding CPT NPs and PDA into a hydrogel matrix crosslinked between CS and aldehyde-functionalized polyethylene glycol (PEG-CBA) (Fig. 4A). PDA was synthesized according to a previously reported method [40], and the synthetic route of PEG-CBA is illustrated in Fig. S7. The appearance of characteristic proton signals at 8.23 ppm and 7.96 ppm in the ^1^H NMR spectrum (Fig. S8), corresponding to aromatic protons, indicated the successful synthesis of PEG-CBA. The formation of CPT NPs gel was visually verified by the vial inversion method, as evidenced by the absence of flow upon tube inversion (Fig. 4B). CPT NPs gel exhibited excellent injectability, as it could be smoothly extruded from a 22G syringe needle. Remarkably, a predefined shape spelling “GEL” was precisely formed upon injection (Fig. S9). This property permits the direct administration of the hydrogel loaded with functional components into surgical sites, facilitating localized delivery and in situ therapeutic intervention.

CPT NPs gel is anticipated to exhibit favorable self-healing behavior owing to its dynamic imine bond-based cross-linked network. The macroscopic self-repair ability was evaluated on a cell culture dish, where an artificially introduced puncture closed completely within 1 min at room temperature, evidencing rapid recovery of the gel network (Fig. 4C). The rheological properties of CPT NPs gel were further investigated by strain sweep (Fig. 4E) and frequency sweep (Fig. 4F). Under a fixed angular frequency of 1 rad/s, strain sweep analysis was performed to evaluate the mechanical resilience of the gel network. As the strain increased from 1 % to 1000 %, the gel initially exhibited elastic-like behavior, characterized by a storage modulus (G′) consistently higher than the loss modulus (G″). When the strain reached the critical strain point of approximately 400 %, G′ intersected with G″, indicating structural disruption and a transition to a viscous-dominated state. In the frequency sweep analysis, G′ remained higher than G″ across the entire frequency range, confirming the predominantly elastic nature and stability of the dynamic cross-linked network. Such self-healing capacity, attributed to the dynamic imine bond-based cross-linking, enables rapid network reconstruction following mechanical damage. This adaptive property allows the gel to conformally fill irregular surgical defects, thereby enhancing localized therapeutic delivery and promoting tissue integration.

The cross-sectional morphology of the freeze-dried gel was characterized using scanning electron microscopy (SEM) (Fig. 4D). The hydrogels exhibited a three-dimensional honeycomb-like porous network. Compared with CS gel and free CPT gel, both PDA gel and CPT NPs gel displayed a more homogeneous pore arrangement and reduced pore dimensions, potentially arising from the increased crosslinking density induced by the presence of PDA [46]. CLSM was employed to perform z-stack imaging of the hydrogel (Fig. 4G). CPT fluorescence appeared as discrete puncta evenly dispersed within the hydrogel matrix, confirming the uniform incorporation of CPT NPs throughout the gel network.

The photothermal performance of CPT NPs gel was evaluated under 808 nm near-infrared (NIR) laser irradiation (1 W/cm^2^). As shown in the thermal images (Fig. 4H), PDA-containing hydrogels (1 mL) exhibited marked temperature elevation upon laser exposure, with the heating rate positively correlated with PDA concentration. Notably, the photothermal effect reached a plateau when the PDA concentration increased from 25 mg/mL to 35 mg/mL. Therefore, CPT NPs gel containing 25 mg/mL PDA was chosen for subsequent studies. In contrast, CS gel showed negligible temperature change under laser irradiation. CPT NPs gel displayed a rapid temperature rise from 25 °C to 59.5 °C within 6 min (Fig. 4I). Meanwhile, the temperature elevation of the hydrogel exhibited a positive correlation with the laser power density (Fig. S10). The hydrogel reached 46.3 °C within 2 min under 1 W/cm^2^ irradiation, which is sufficient to induce a photothermal effect for tumor ablation (above 45 °C required) [47]. Under five consecutive on-off laser irradiation cycles (10 min irradiation), CPT NPs gel maintained a stable photothermal response with a reproducible temperature elevation (*Δ*T ≈ 44 °C) during each irradiation period (Fig. 4J), demonstrating its robust and repeatable photothermal stability. In addition, the photothermal conversion efficiency (η) of CPT NPs gel was calculated to be 46.6 % based on the heating-cooling curve and the corresponding −ln(θ) versus time plot (Fig. S11), following the method reported in the literature [48].

To investigate the degradation-mediated release behavior, CPT NPs gel was incubated in PBS for 48 h. The resulting degradation medium was concentrated and examined by TEM (Fig. 4K), which revealed abundant spherical NPs with an average diameter of 49.1 nm (PDI: 0.16), consistent with the size of the initially embedded CPT NPs. This observation suggests that CPT NPs were gradually liberated from the hydrogel matrix during the degradation process. In vivo biodegradation was further assessed by subcutaneous implantation of the gel into BALB/c nude mice (Fig. 4L). The degradation profile exhibited a near-linear trend over time, leaving 22.8 % of the gel mass after 28 days. This sustained degradation behavior supports the potential of CPT NPs gel to serve as a long-acting depot for the prolonged release of CPT NPs.

Motivated by the excellent photothermal performance and degradation-mediated drug release, we next evaluated the in vitro antitumor efficacy of the gels using the CCK-8 assay (Fig. 4M). Both CS gel and PDA gel exhibited minimal cytotoxicity against MuM-2B cells. In contrast, treatment with free CPT gel and CPT NPs gel decreased cell viability to 64.8 % and 73.8 %, respectively, which was attributed to the cytotoxic effects of CPT or CPT NPs released during gel degradation. Upon treatment with CPT NPs gel followed by 808 nm laser irradiation (1 W/cm^2^), the cell viability dramatically decreased to 13.2 %, markedly lower than that of the PDA gel + NIR group, whereas other control groups exhibited no significant changes in cell viability upon laser exposure, demonstrating the potent synergistic photothermal-chemotherapy efficacy of CPT NPs + NIR in tumor cell ablation. Hyperthermia-induced ablation is inherently non-specific, potentially affecting both tumor and normal cells [49]. Nevertheless, when applied as an adjuvant postoperative therapy, localized photothermal treatment can selectively eradicate residual tumor cells at the surgical site, thereby reducing the risk of recurrence while minimizing systemic side effects associated with thermal exposure.

### 3.5 Preventing postoperative tumor recurrence of nanocomposite hydrogel

**Fig. 5:**
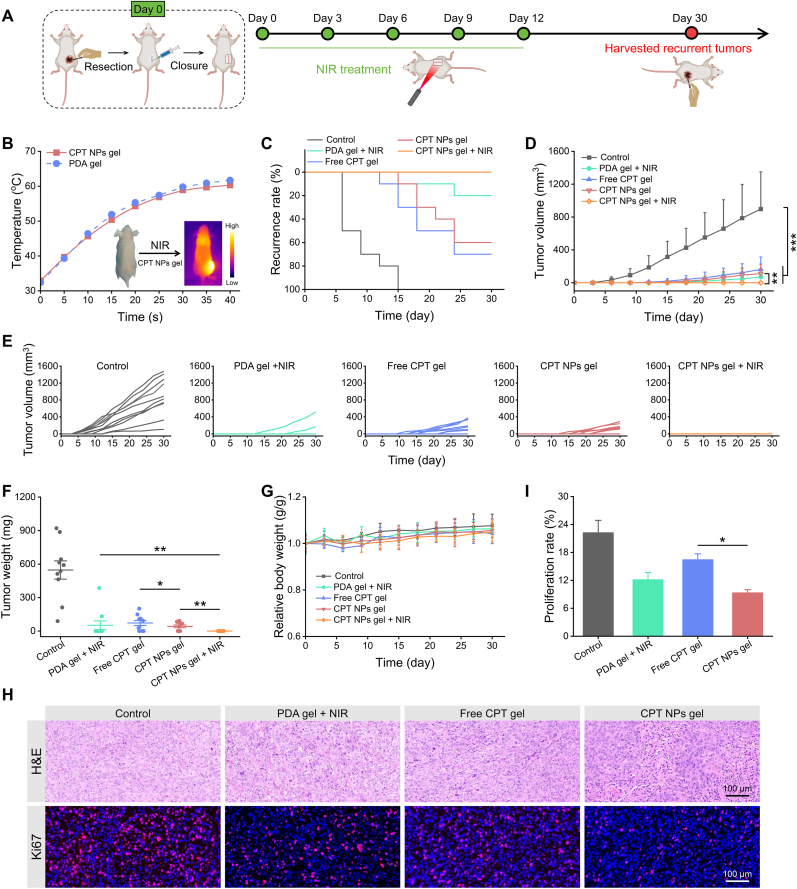
**Preventing postoperative tumor recurrence of nanocomposite hydrogel.** (A) Schematic illustration of anti-recurrence treatment. (B) Temperature variations at the postoperative site of CPT NPs gel and PDA gel under 808 nm laser irradiation (1 W/cm^2^). (C) Incidence of local recurrence in MuM-2B tumors across different treatment groups. (D) Growth curves of recurrent tumors. (E) Individual tumor growth profiles in each treatment group. (F) Recurrent tumor weight in different treatment groups. (G) Body weight changes of mice throughout the treatment period. (H) Representative H&E and Ki67 staining of recurrent MuM-2B tumors. (I) Quantitative analysis of tumor cell proliferation.

To evaluate the anti-recurrence efficacy of CPT NPs gel, a partially resected MuM-2B tumor mouse model was established to mimic clinical scenarios of postsurgical residual tumor cells. This model retains a controlled portion of tumor tissue after surgery, reproducibly simulating postoperative residual lesions. Compared with conventional UM xenograft or full-resection models, this model more faithfully reflects the clinical challenge of incomplete tumor removal, substantially recapitulates key aspects of the postoperative residual tumor microenvironment, and allows direct evaluation of therapeutic efficacy in preventing recurrence [50]. When the tumor volume reached ∼120 mm^3^, approximately 90 % of the tumor burden was surgically removed. Mice were randomly divided into 5 groups (n = 10 per group) and treated at the resection site with a 100 μL injection of PBS (Control), PDA gel + NIR, free CPT gel, CPT NPs gel, or CPT NPs gel + NIR. For the groups receiving photothermal therapy, the surgical bed was irradiated with an 808 nm NIR laser (1 W/cm^2^) for 40 s under anesthesia on days 0, 3, 6, 9, and 12 (Fig. 5A). As shown in Fig. 5B, CPT NPs gel induced a rapid temperature increase at the postoperative site upon NIR irradiation, reaching approximately 60 °C within 40 s, indicative of robust in vivo photothermal conversion. Tumor recurrence, tumor growth, and body weight were monitored throughout the treatment period.

Tumor recurrence was first observed in the control group, where 5 mice developed recurrent tumors on day 6 post-surgery, and all mice relapsed by day 15, resulting in a 100 % recurrence rate (Fig. 5C). In comparison, partial suppression of recurrence was observed in the free CPT gel and CPT NPs gel groups, with 7 and 6 mice relapsing by day 30, respectively (Fig. S12). The PDA gel + NIR group exhibited a pronounced reduction in recurrence, with only 2 mice developing recurrent tumors. Strikingly, no recurrence was detected in the CPT NPs gel + NIR group throughout the entire observation period, highlighting potent anti-recurrence efficacy through synergistic photothermal-chemotherapy. Moreover, recurrent tumors in all drug-treated groups exhibited markedly slower growth compared to the control group (Fig. 5D and E), which was consistent with tumor weights at the experimental endpoint (Fig. 5F). The average tumor weight in the control group reached 546.9 mg, whereas those in the PDA gel + NIR, free CPT gel and CPT NPs gel groups were substantially reduced to 51.8 mg, 72.4 mg and 42.3 mg, respectively. Histological analysis of recurrent tumors revealed extensive nuclear fragmentation in the CPT NPs gel group (Fig. 5H). In addition, Ki-67 immunofluorescence staining demonstrated a marked reduction in red fluorescence intensity, suggesting suppressed proliferative activity. Quantitative analysis using ImageJ software showed a proliferation rate of 9.3 % in the CPT NPs gel group, significantly lower than the 16.4 % in the free CPT gel group (Fig. 5I). This enhanced antiproliferative effect was attributed to improved cellular uptake and tumor-targeted accumulation of CPT NPs relative to free CPT. No significant body weight loss or abnormal behavior was observed in any group throughout the treatment period (Fig. 5G). The underlying mechanisms responsible for the potent anti-recurrence effect observed in the CPT NPs gel + NIR group can be attributed to the synergistic combination of photothermal and chemotherapeutic effects. Rapid local hyperthermia induced by NIR irradiation causes irreversible damage to residual tumor tissues, while the sustained release of charge-reversal CPT NPs enhances cellular uptake and facilitates eradication of dispersed tumor cells. Although the therapeutic outcome may be influenced by factors such as tumor model, laser power, irradiation area, and combination of therapeutic agents, this strategy remains a highly promising and effective approach for postoperative management of uveal melanoma.

### 3.6 Inhibition of tumor metastasis

**Fig. 6:**
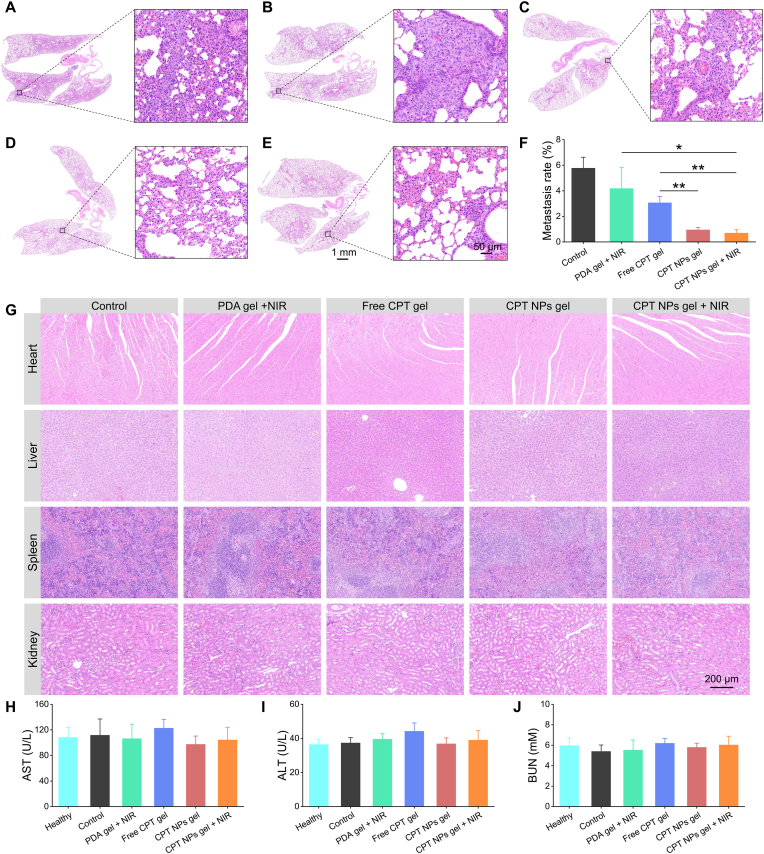
**Inhibition of tumor metastasis.** H&E-stained lung sections collected on day 30 from the (A) control group, (B) PDA gel + NIR group, (C) free CPT gel group, (D) CPT NPs gel group, and (E) CPT NPs gel + NIR group. (F) Quantified pulmonary metastasis rates of different treatment groups based on H&E-stained lung sections. (G) H&E staining of major organs (heart, liver, spleen, kidney) from each group. Levels of (H) AST, (I) ALT, and (J) BUN in healthy mice and post-treatment mice.

Upon completion of postoperative treatment, major organs were harvested for histological analysis using H&E staining to assess tumor metastasis. Lung sections from the control group exhibited extensive pulmonary metastases, confirming the highly aggressive metastatic nature of UM (Fig. 6A). Although PDA gel + NIR treatment effectively suppressed local tumor recurrence, substantial tumor cell infiltration was still detected in the lungs (Fig. 6B), likely resulting from intraoperative dissemination of tumor cells into the bloodstream. In contrast, lungs from mice treated with free CPT gel, CPT NPs gel, and CPT NPs gel + NIR displayed markedly reduced tumor infiltration (Fig. 6C–E), underscoring the benefit of chemotherapy in mitigating UM metastasis. The metastatic burden was further quantified by calculating the percentage of lung area occupied by infiltrating tumor cells (Fig. 6F). Notably, the CPT NPs gel and CPT NPs gel + NIR groups showed significantly lower pulmonary metastasis rates of 0.93 % and 0.67 %, respectively, compared with 3.1 % in the free CPT gel group. This reduction can arise from the enhanced tumor-targeting capability of the released CPT NPs, which improved chemotherapeutic efficacy. No evidence of tumor infiltration or structural damage was detected in the heart, liver, spleen, or kidneys in any treatment group (Fig. 6G). Moreover, serum levels of aspartate aminotransferase (AST, Fig. 6H), alanine aminotransferase (ALT, Fig. 6I), blood urea nitrogen (BUN, Fig. 6J), and creatinine (CREA, Fig. S13) remained within the normal physiological range. Skin tissues adjacent to the surgical resection site in the CPT NPs gel + NIR group were collected on day 30 and analyzed by H&E staining, TUNEL and CD68 immunofluorescence (Fig. S14). The results revealed intact tissue structure, negligible apoptosis, and no appreciable abnormal macrophage infiltration, indicating that the applied localized photothermal therapy and chemotherapy did not cause significant damage to the surrounding healthy tissue or systemic toxicity.

Interestingly, despite the well-documented clinical tendency of UM to metastasize to the liver [4], pulmonary metastasis was predominant in our study. This difference can be attributed to several model-specific factors: (i) xenograft models in immunodeficient nude mice cannot fully recapitulate the native tumor microenvironment associated with metastasis, the residual innate immunity and the presence of NK cells may suppress spontaneous liver metastasis; (ii) intraoperative dissemination of tumor cells into systemic circulation, with the highly vascularized pulmonary capillary network serving as an initial filtration site in mice, favoring lung colonization; (iii) inoculation sites can influence metastatic patterns, as tumor cells display tissue-dependent differences in invasiveness, angiogenesis, and stromal interactions [[51], [52], [53]]. Additionally, published genetic mouse models of UM frequently develop pulmonary rather than hepatic metastases, underscoring interspecies differences in metastatic biology [54]. Taken together, the observed suppression of lung metastasis in our study reflects a reduction in metastatic potential rather than a direct replication of clinical UM metastasis. Although this constitutes a limitation of the model, these findings provide supportive preclinical evidence that the proposed therapeutic strategy can effectively inhibit tumor dissemination, highlighting its potential significance for developing interventions to prevent postoperative metastasis in UM.

### 3.7 In vivo safety evaluation

To further evaluate the safety of the CPT NPs gel + NIR treatment system, CPT NPs gel was subcutaneously injected into healthy Balb/c nude mice, followed by laser irradiation under the same parameters as in the anti-recurrence study. Body weight of all mice steadily increased during the treatment period (Fig. S15). On day 21, serum biochemical parameters, including alanine aminotransferase (ALT), aspartate aminotransferase (AST), blood urea nitrogen (BUN), creatinine (CREA), creatine kinase (CK), lactate dehydrogenase (LDH), total cholesterol (TC), triglycerides (TG) and glucose (GLU), were measured (Fig. S16). No significant differences were observed compared with untreated healthy mice, indicating that the treatment did not induce acute or chronic toxicity to cardiac, hepatic, or renal function. Furthermore, H&E staining of major organs revealed no abnormalities or pathological lesions (Fig. S17), and histological and immunohistochemical analyses confirmed that the hydrogel caused no damage to the surrounding skin (Fig. S18). Collectively, these findings demonstrate the favorable in vivo biosafety of the CPT NPs gel + NIR system.

## 4 Conclusions

In conclusion, we successfully developed a CS-based injectable multi-responsive nanocomposite hydrogel (CPT NPs gel), co-encapsulating pH/GSH-responsive CPT NPs and the photothermal agent PDA for synergistic photothermal-chemotherapy to prevent postoperative recurrence and metastasis of UM. The CPT NPs gel displayed a three-dimensional honeycomb-like porous network, demonstrating excellent injectability and self-healing properties. It was capable of persisting in vivo for over 28 days, gradually degrading while sustainably releasing CPT NPs. The CPT NPs remained stable under physiological conditions but underwent a charge reversal from −7.4 mV to +14.9 mV in response to the acidic tumor microenvironment, which enhanced cellular uptake. Once exposed to elevated intracellular GSH levels, the NPs disassembled and rapidly liberated CPT, resulting in potent inhibition of tumor cell proliferation, invasion, and migration. Upon postoperative administration, the CPT NPs gel rapidly heated to approximately 60 °C within 40 s under 808 nm laser irradiation (1 W/cm^2^), effectively abrogating local tumor recurrence. Histological analysis revealed that treatment with the CPT NPs gel significantly reduced the risk of distant lung metastasis, with pulmonary metastatic infiltration areas remaining below 1 %, a marked reduction compared to other treatment groups, while no evident systemic toxicity was detected. Consequently, the CPT NPs gel embodies a promising intelligent therapeutic strategy with substantial potential to improve the prognosis of UM patients by reliably preventing postoperative recurrence and metastasis.

## CRediT authorship contribution statement

**Zhihao Guo:** Writing – original draft, Project administration, Methodology, Investigation, Funding acquisition, Formal analysis, Data curation, Conceptualization. **Jiangcheng Tan:** Investigation, Visualization, Validation, Supervision, Software, Methodology, Formal analysis, Data curation. **Junjie Chen:** Methodology, Formal analysis. **Jieqiong Liu:** Methodology. **Wei Xiao:** Visualization, Validation, Supervision, Methodology. **Xinyuan Huang:** Validation, Methodology. **Cailing Wei:** Methodology. **Ruohua Zhu:** Software, Formal analysis. **Ji-Liang Li:** Writing – review & editing, Resources, Funding acquisition, Formal analysis, Conceptualization.

## Declaration of competing interest

The authors declare that they have no known competing financial interests or personal relationships that could have appeared to influence the work reported in this paper.

## Data Availability

Data will be made available on request.
