# Supplementary material for: Multi-responsive nanocomposite hydrogel for synergistic photothermal-chemotherapy to prevent postoperative recurrence and metastasis of uveal melanoma
Source: Mater Today Bio. 2025 Dec 3;35:102628. doi: 10.1016/j.mtbio.2025.102628 (PMC12731282; doi:10.1016/j.mtbio.2025.102628)
Supplement: Multimedia component 1 [file mmc1.docx]

*Supporting information*

**Multi-responsive nanocomposite hydrogel for synergistic photothermal-chemotherapy to prevent postoperative recurrence and metastasis of uveal melanoma**

Zhihao Guo^a, 1,^ *, Jiangcheng Tan^a, 1^, Junjie Chen^c^, Jieqiong Liu^a^, Wei Xiao^a^, Xinyuan Huang^a^, Cailing Wei^a^, Ruohua Zhu^a^, Ji-Liang Li ^a, b,^ **

1. National Engineering Research Center of Ophthalmology and Optometry, School of Biomedical Engineering, Eye Hospital, Wenzhou Medical University, Wenzhou, 325027, China
2. Wenzhou Institute, University of Chinese Academy of Sciences, Wenzhou, 325000, China
3. Hangzhou GreenIce EcoTech Co., Ltd., Hangzhou, 311200, China

***Corresponding author**

E-mail for Zhihao Guo: [guozh@wmu.edu.cn](mailto:guozh@wmu.edu.cn)

E-mail for Ji-Liang Li: [jlilab1971@wmu.edu.cn](mailto:jlilab1971@wmu.edu.cn) (ORCID: [0000-0001-6200-1007](https://orcid.org/0000-0001-6200-1007))

^1^ These authors contributed equally to this work.

## Experimental Section

## *Materials*

Camptothecin, triphosgene, and bis(2-Hydroxyethyl) disulfide were purchased from Adamas-beta Co., Ltd. (Shanghai, China). Chitosan (CS, 100−200 mpa.s), poly(ethylene glycol) methyl ether (mPEG_113_), polyethylene glycol (*M*_n_: 4000), 4-formylbenzoic acid, N-(2-hydroxyethyl) hexamethyleneimine (NHE), dopamine hydrochloride, 1-(3-dimethylaminopropyl)-3-ethylcarbodiimide (hydrochloride) (EDCI), N,N’-dicyclohexyl carbodiimide (DCC), 4-dimethylaminopyridine (DMAP), 1,8-diazabicyclo[5.4.0]undec-7-ene (DBU), benzoic acid, dimethyl sulfoxide (DMSO), dichloromethane (DCM), tetrahydrofuran (THF), were purchased from Aladdin Industrial Co., Ltd. (Shanghai, China). Palladium 10% on carbon (Pd/C) and palladium hydroxide (Pd(OH)_2_/C) were purchased from Beijing InnoChem Science & Technology Co., Ltd. (Beijing, China). Silica gel (300–450 m²/g, 50–75 μm) was purchased from Qingdao Haiyang Chemical Co., Ltd. (Qingdao, China). Cell counting kit-8 (CCK-8) were purchased from Dalian Meilun Biotechnology Co., Ltd. (Dalian, China). Matrigel, 4% formaldehyde solution, crystal violet were purchased from Beijing Solarbio Technology Co. Ltd. (Beijing, China). Roswell Park Memorial Institute (RPMI)-1640 medium and fetal bovine serum were purchased from Life Technologies Corporation (USA). 5-Methyl-5-benzyloxycarbonyl-1,3-dioxan-2-one (MBC) was synthesized as our previous report [1].

## *Characterization of mPEG-PMCC-(PMCC-CPT)*

The structures of polymers were characterized by ^1^H NMR (Bruker Avance II-400 MHz, Bruker) and FT-IR (PerkinElmer spectrum One, PerkinElmer). The dispersity was determined using a gel permeation chromatographic (GPC) system (Waters 1515, USA).

GSH-triggered degradation of mPEG-PMCC-(PMCC-CPT): To evaluate the degradation of mPEG-PMCC-(PMCC-CPT), 1 mL of mPEG-PMCC-(PMCC-CPT) solution (2 mg/mL) was transferred into a dialysis bag (MWCO: 3500 Da), which was then immersed in DMSO containing 10 mM GSH and incubated at room temperature under gentle agitation. At predetermined time intervals, the mPEG-PMCC-(PMCC-CPT) solution in the dialysis bag was quantified to a final volume of 1 mL, and the absorbance was measured using a Cary 5000 UV-Vis-NIR (Agilent, USA).

## *Characterization of CPT-loaded NPs*

Morphology and size of NPs: The morphology of the prepared NPs was characterized using transmission electron microscopy (TEM, FEI Tecnai 12, USA), while their hydrodynamic diameter and size distribution were analyzed with a Zetasizer Nano ZS/ZEN3690 (Malvern, England).

Drug loading content (LC): The CPT NPs were lyophilized and subsequently redissolved in 1,4-dioxane. The absorbance of the resulting solution at 365 nm was measured using a Cary 5000 UV-Vis-NIR (Agilent, USA), and the LC was calculated according to a calibration curve.

Stability of NPs: The stability of CPT NPs was assessed by monitoring changes in particle size and zeta potential in PBS supplemented with 10% FBS at pH 7.4. The NPs were gently mixed with the serum-containing medium and incubated under physiological conditions. At predetermined time intervals, the particle size and zeta potential were measured using a Zetasizer Nano ZS/ZEN3690 (Malvern, England). All measurements were performed in triplicate to ensure accuracy and reproducibility.

GSH-responsive drug release: The GSH-responsive drug release behavior of CPT NPs was evaluated using a dialysis method. Specifically, 2 mL of CPT NPs solution (CPT concentration: 1 mg/mL) was loaded into a dialysis bag (MWCO: 3500 Da) and immersed in 25 mL of PBS containing 10 mM GSH. The system was maintained at 37  ℃ with gentle agitation at 120 rpm in the dark. At predetermined time points, 5 mL of the external medium was withdrawn for CPT concentration analysis and immediately replaced with an equal volume of fresh PBS. The cumulative drug release was then calculated based on the concentration measurements. The solution inside the dialysis bag was collected and concentrated by ultrafiltration, the morphology and size of the NPs were subsequently examined.

## *Characterization of nanocomposite hydrogel*

Self-healing of CPT NPs gel: CPT NPs gel was placed on a cell culture dish, and a hole approximately 1 cm in diameter was created at the center of the hydrogel. The closure of the hole was observed, and the time required for the hydrogel to fully recover was recorded. The rheological behavior of CPT NPs gel was measured using a Haake Mars40 (Thermo Scientific).

Photothermal performance: Temperature changes of CPT NPs gel under near-infrared (NIR) irradiation (808 nm, 1 W/cm²) were monitored using an infrared thermal imager. CPT NPs gel (1 mL) was placed in a 5 mL vial, and the laser was irradiated from the top of the vial. The temperature and thermal images of the hydrogel were captured from the side of the vial. Additionally, the temperature variations of CPT NPs gel under different laser power densities (0.5, 1 and 1.5 W/cm^2^) were further evaluated.

Release CPT NPs: 1 mL of CPT NPs gel was soaked in 3 mL of PBS, the system was gently shaken at a speed of 120 rpm under 37 °C for 48 h. The supernatant was collected for TEM observation.

In vivo degradation of CPT NPs gel: CPT NPs gel (100 μL) was subcutaneously injected into the left lower backs of mice. At predetermined time intervals, the residual hydrogels were surgically excised, photographed, and immediately weighed to determine their wet weight.

## *Cellular uptake*

MuM-2B cells were plated into glass-bottomed dishes at a density of 1.5×10^5^ cells per well and incubated for 12 h in a humidified atmosphere of 5% CO_2_ at 37 °C. Afterward, the original medium was replaced with a CPT-loaded NPs-containing medium (10 μg/mL equivalent CPT concentration) and incubated for another 6 h. The medium was then removed, and the cells were fixed with 4% paraformaldehyde solution for 15 min. The samples were observed using confocal laser scanning microscopy (CLSM, Zeiss LSM 880, Germany) with excitation at 405 nm and emission detected between 425 and 475 nm.

## *In vitro cytotoxicity*

To evaluate the cytotoxicity of the drug solutions, MuM-2B cells were seeded in 96-well plates at a density of 5×10³ cells per well and allowed to adhere for 12 h. Subsequently, cells were treated with various concentrations of different drug formulations. After 24 h of incubation, the medium was replaced with fresh medium containing CCK-8 reagent and incubated for an additional 2 h. Absorbance at 450 nm was measured using a microplate reader. Cell viability was expressed as the percentage of absorbance relative to that of untreated control cells.

For hydrogel formulations, MuM-2B cells were seeded in 24-well plates at a density of 2×10⁴ cells per well and incubated for 12 h. Subsequently, 100 μL of hydrogel (equivalent to 1 mg/mL CPT) was added into transwell inserts and placed into the wells containing culture medium. After 24 h of co-incubation, the culture medium was replaced with serum-free RPMI-1640 medium containing CCK-8 reagent. Following an additional 2 h incubation, absorbance at 450 nm was recorded using a microplate reader. Cell viability was calculated as the percentage of absorbance relative to the untreated control group.

For in vitro PTT: MuM-2B cells were seeded in 96-well plates at a density of 5×10³ cells per well and allowed to adhere for 12 h. Subsequently, 40 μL of CPT NPs gel was administered to each well, and wells without gel treatment served as the control group. For in vitro PTT, cells treated with CPT NPs gel were exposed to 808 nm laser irradiation (1 W/cm²) for 5 min. Following irradiation, cells were incubated for an additional 24 h, after which cell viability was assessed.

## *In vitro anti-metastasis effect*

In vitro anti-metastasis effect of CPT NPs was evaluated by wound healing assay and transwell migration assay. For the wound healing assay, MuM-2B cells were seeded in 6-well plates at a density of 4 × 10⁵ cells per well and cultured until reaching approximately 90% confluence. A linear wound was introduced by scratching the cell monolayer with a sterile 10 μL pipette tip. After removing cell debris by gently washing with PBS, the medium was replaced with RPMI-1640 containing 0.1% FBS and either free CPT or CPT NPs (equivalent CPT concentration: 0.1 μg/mL). The cells were then incubated for 24 h. Wound closure was monitored by capturing images of the wound area at 0 and 24 h. The wound areas were analyzed using ImageJ software, and the wound healing rate was calculated as the percentage of wound healing area compared to the initial wound area.

For the transwell migration assay, MuM-2B cells were pretreated with free CPT or CPT NPs (equivalent to 0.1 μg/mL CPT) in RPMI-1640 medium supplemented with 0.1% FBS for 24 h. Following treatment, the cells were harvested, resuspended, and seeded into the upper chambers of 24-well transwell inserts at a density of 5 × 10⁴ cells per well. The lower chambers were filled with medium containing 10% FBS as a chemoattractant. After 24 h of incubation, non-migrated cells on the upper membrane surface were gently removed with a cotton swab, while the migrated cells on the underside were fixed with 4% paraformaldehyde and stained with 0.1% crystal violet. The stained cells were imaged under a microscope and subsequently eluted with 33% acetic acid for quantification by measuring absorbance at 570 nm.

## *In vitro anti-invasion effect*

The cell invasion assay was conducted following the same protocol as the migration assay, with the exception that the transwell inserts were pre-coated with 60 μL of matrigel on the upper surface of the membrane prior to cell seeding.

## *In vivo anti-recurrence assessment*

To establish a subcutaneous xenograft model, MuM-2B cells (3 × 10⁶ cells suspended in 100 μL of PBS) were subcutaneously injected into right lower backs of BALB/c nude mice, and the tumor volume was measured using a vernier caliper every day. The tumor volume was calculated by the following formula: V = 0.5 × L × W^2^ (L, tumor length; W, tumor width). Mice were euthanized when the tumor volume exceeded 1000 mm^3^ or signs of health impairment were observed. Once tumors reached approximately 150 mm^3^, surgical resection of the tumors was performed, and the overlying and surrounding skin tissues were preserved. Mice were then randomly divided into five groups (10 mice per group): (1) untreated control, (2) PDA gel + NIR irradiation, (3) free CPT gel, (4) CPT NPs gel, and (5) CPT NPs gel + NIR irradiation. Each mouse received a 100 μL injection of the corresponding hydrogel formulation. For NIR-treated groups, mice were irradiated with an 808 nm laser (1.0 W/cm²) for 40 s under anesthesia at two-day intervals, for a total of 5 sessions. The tumor recurrence and body weight of mice were monitored every three days, and the time at which a subcutaneous nodule could be detected was defined as the time of local recurrence. On day 30, all mice were euthanized and the recurrent tumors were harvested for H&E and Ki67 staining analysis.

## *In vivo anti-metastasis assessment*

At the end of the experiment, the heart, liver, spleen, lungs, and kidneys of the mice were harvested, fixed, sectioned, and stained with H&E. Additionally, blood samples were collected for biochemical analysis. Tumor cell infiltration in each organ was examined histologically. The lung metastasis rate was calculated as the ratio of the tumor-infiltrated area to the total lung tissue area in the histological sections.

## *In vivo safety assessment*

CPT NPs gel was injected into the right lower back of healthy female Balb/c nude mice, with the day of injection defined as day 0. Mice were irradiated with an 808 nm laser (1.0 W/cm²) for 40 seconds under anesthesia at two-day intervals, for a total of five sessions, as described in the anti-recurrence section. On day 21, serum samples were collected for evaluation of biochemical indicators, and major organs and the skin adjacent to the irradiation site were harvested for histological examination.


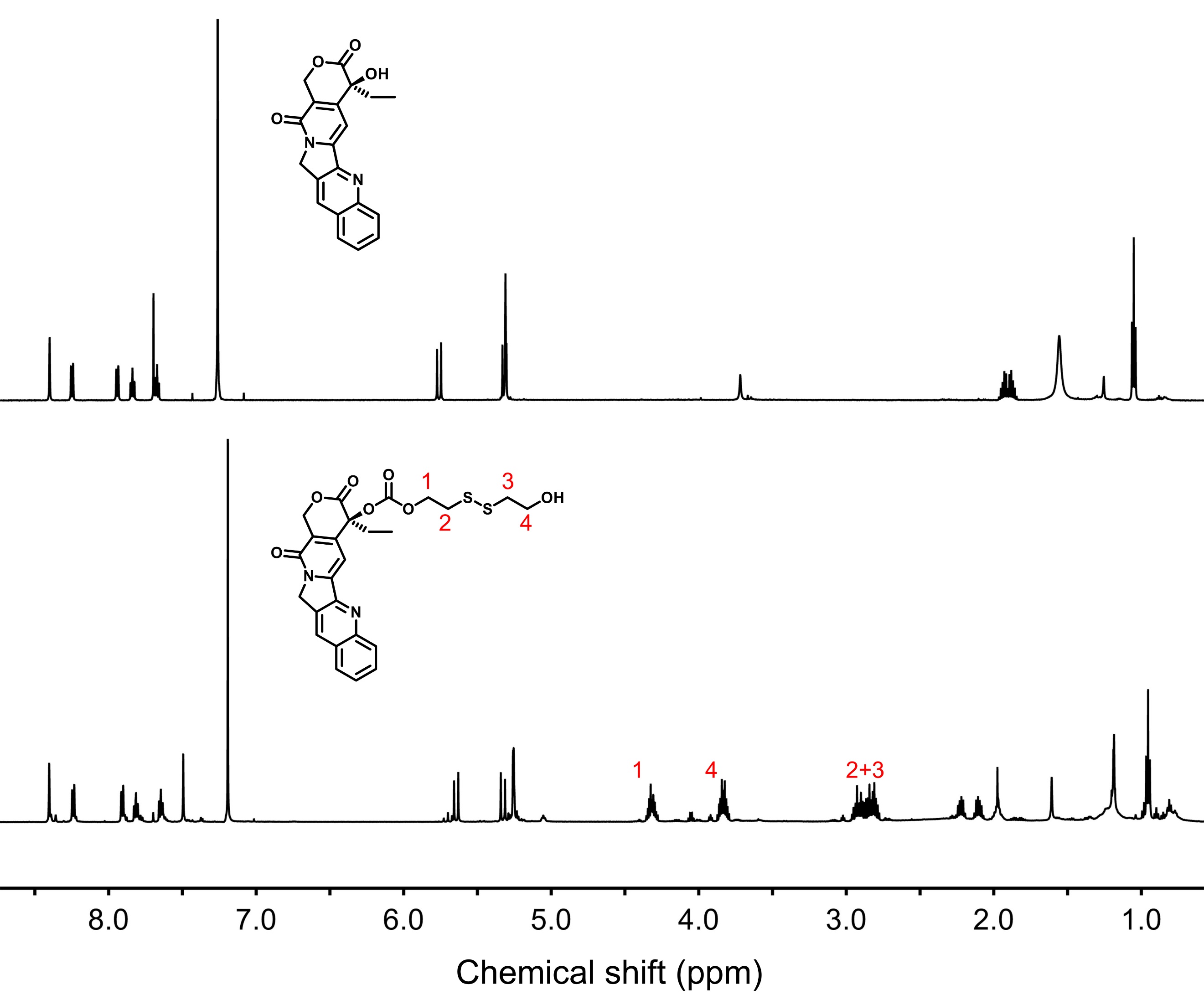


**Figure S1.** ^1^H NMR spectra of CPT and CPT-SS-OH.


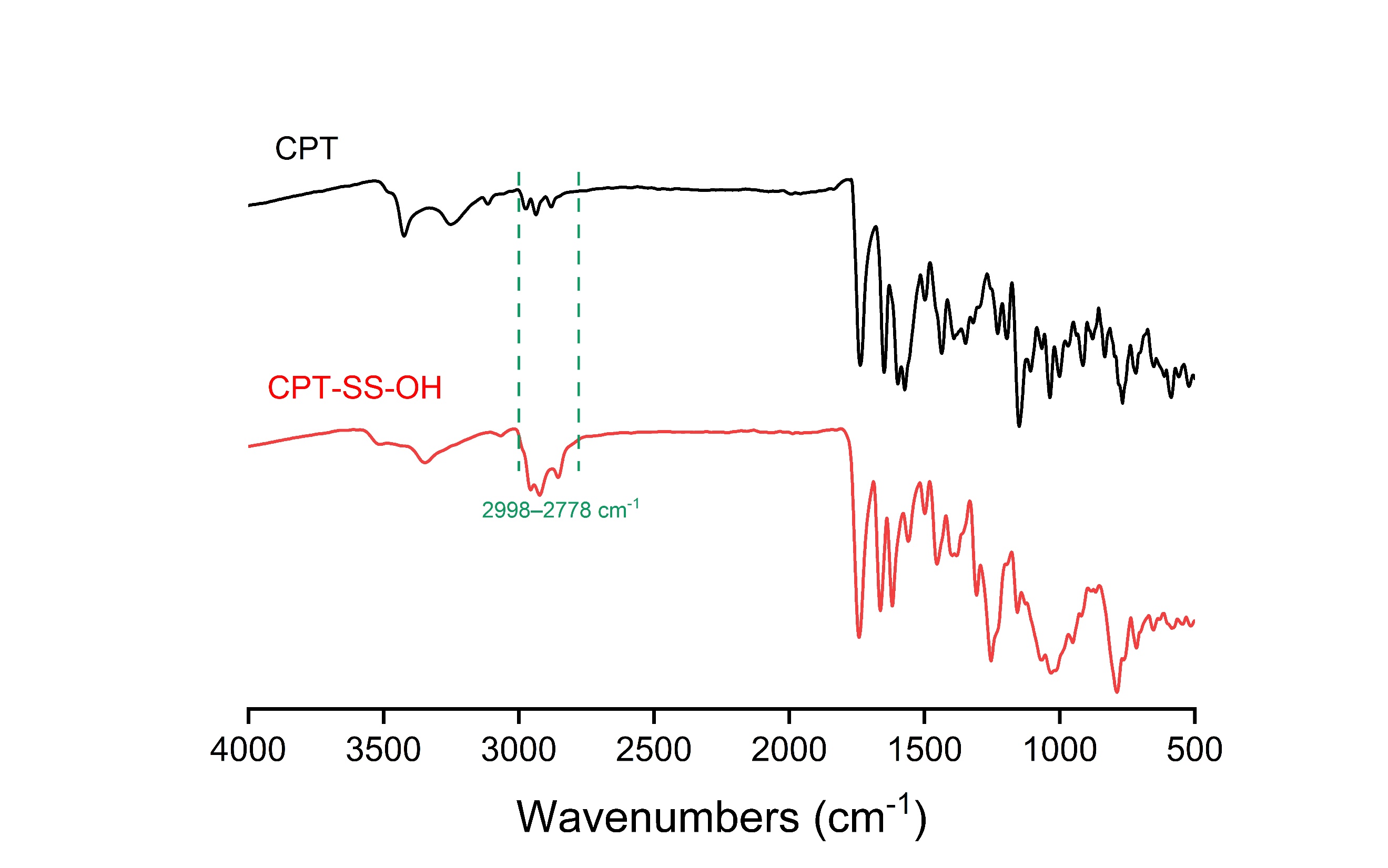


**Figure S2.** FT-IR spectra of CPT and CPT-SS-OH.


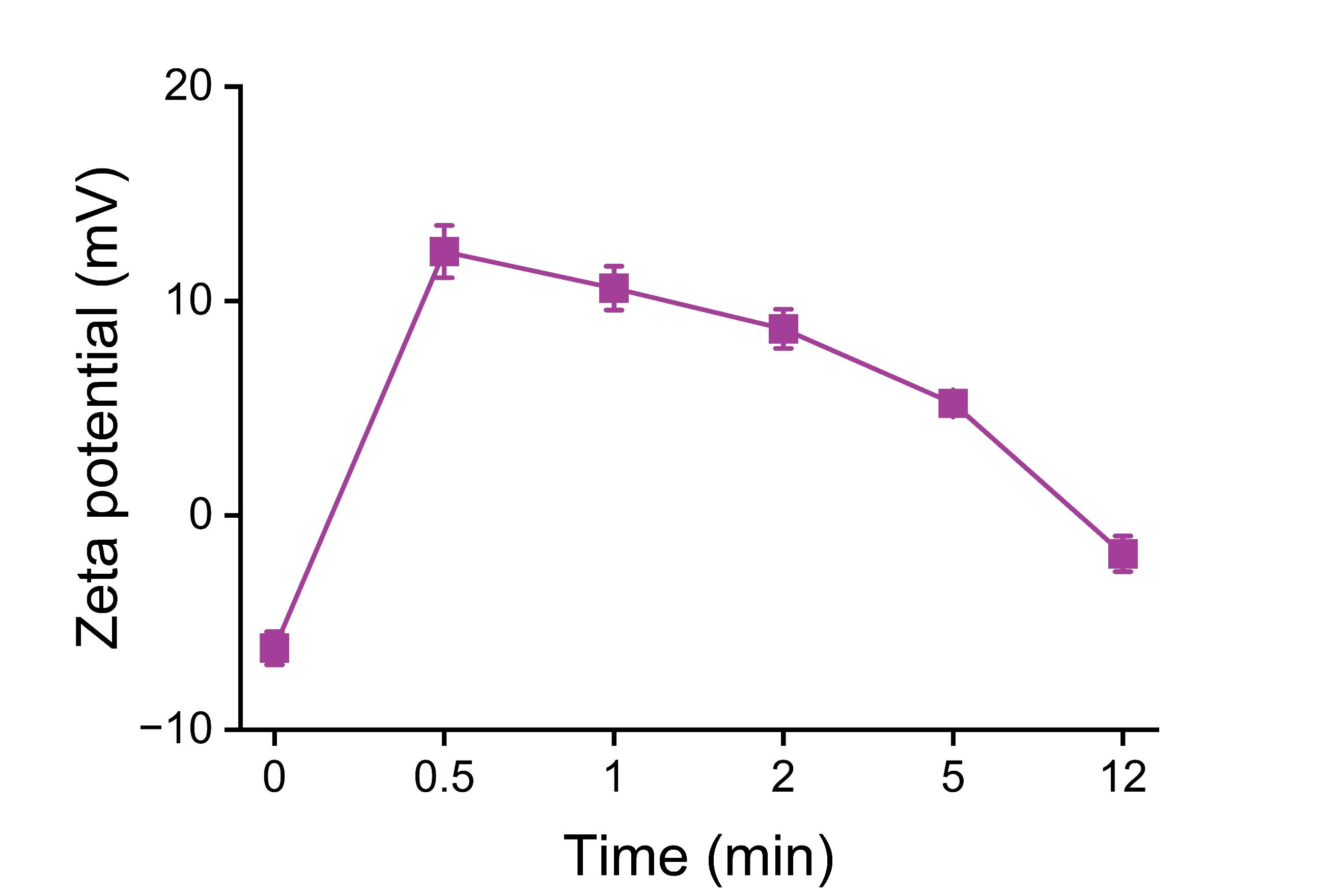


**Figure S3.** Zeta potential of CPT NPs after incubation in PBS at pH 6.5 containing 10% FBS.


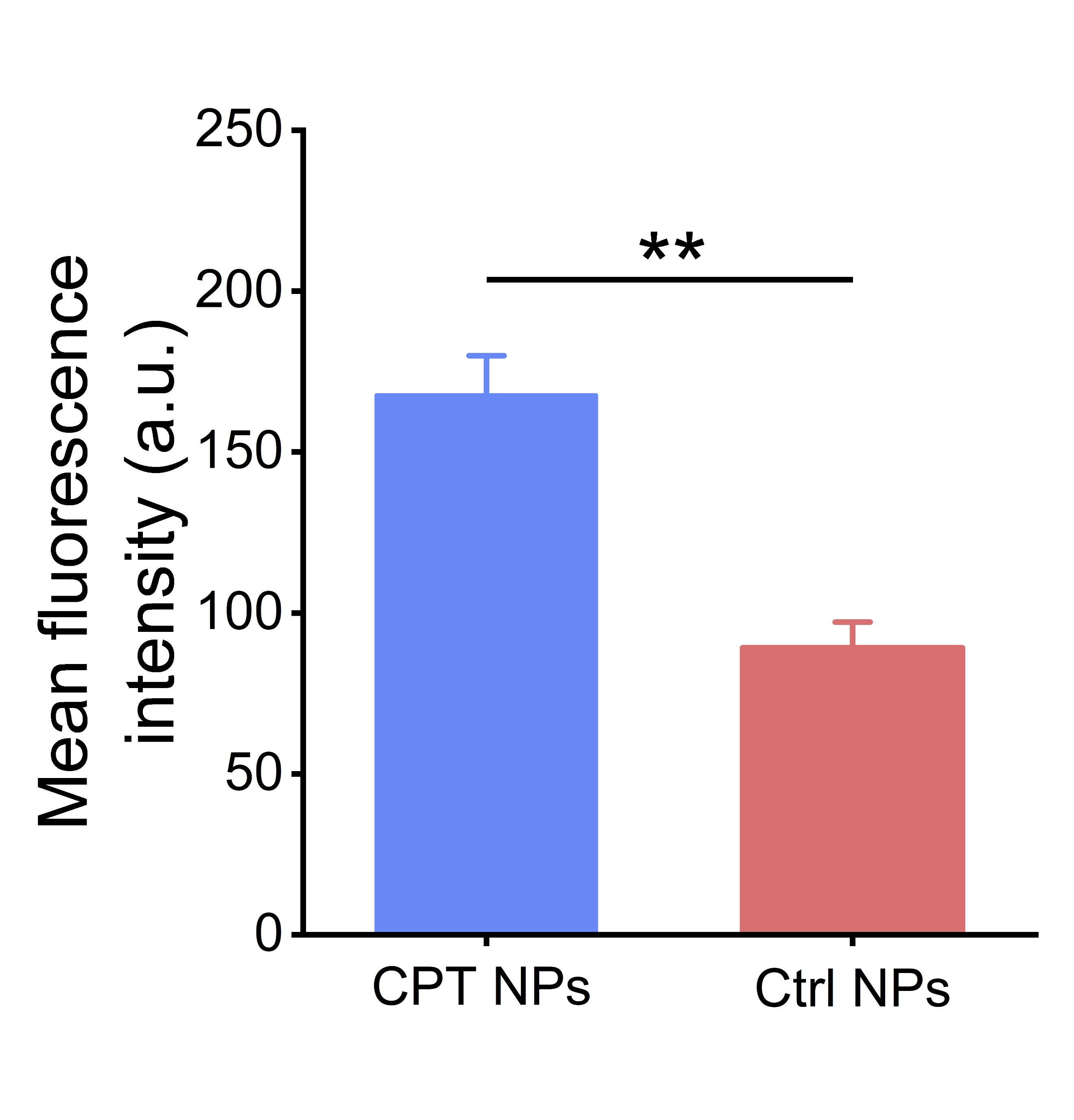


**Figure S4.** Quantitative analysis of CPT fluorescence intensity in MuM-2B cells at pH 6.5 from the CLSM results.


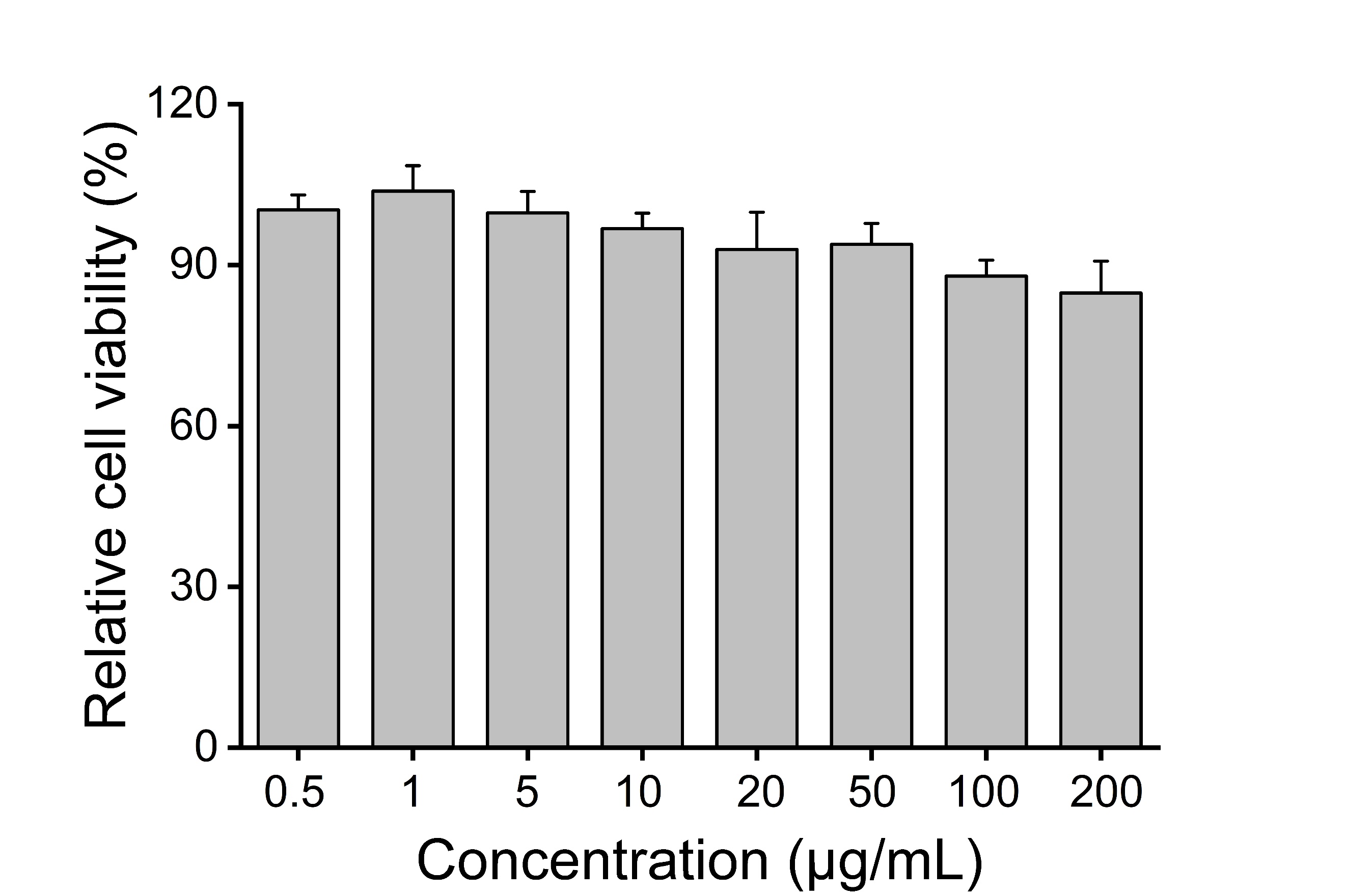


**Figure S5.** Relative cell viability of MuM-2B cells after 24 h incubation with mPEG-*b*-PMCC.


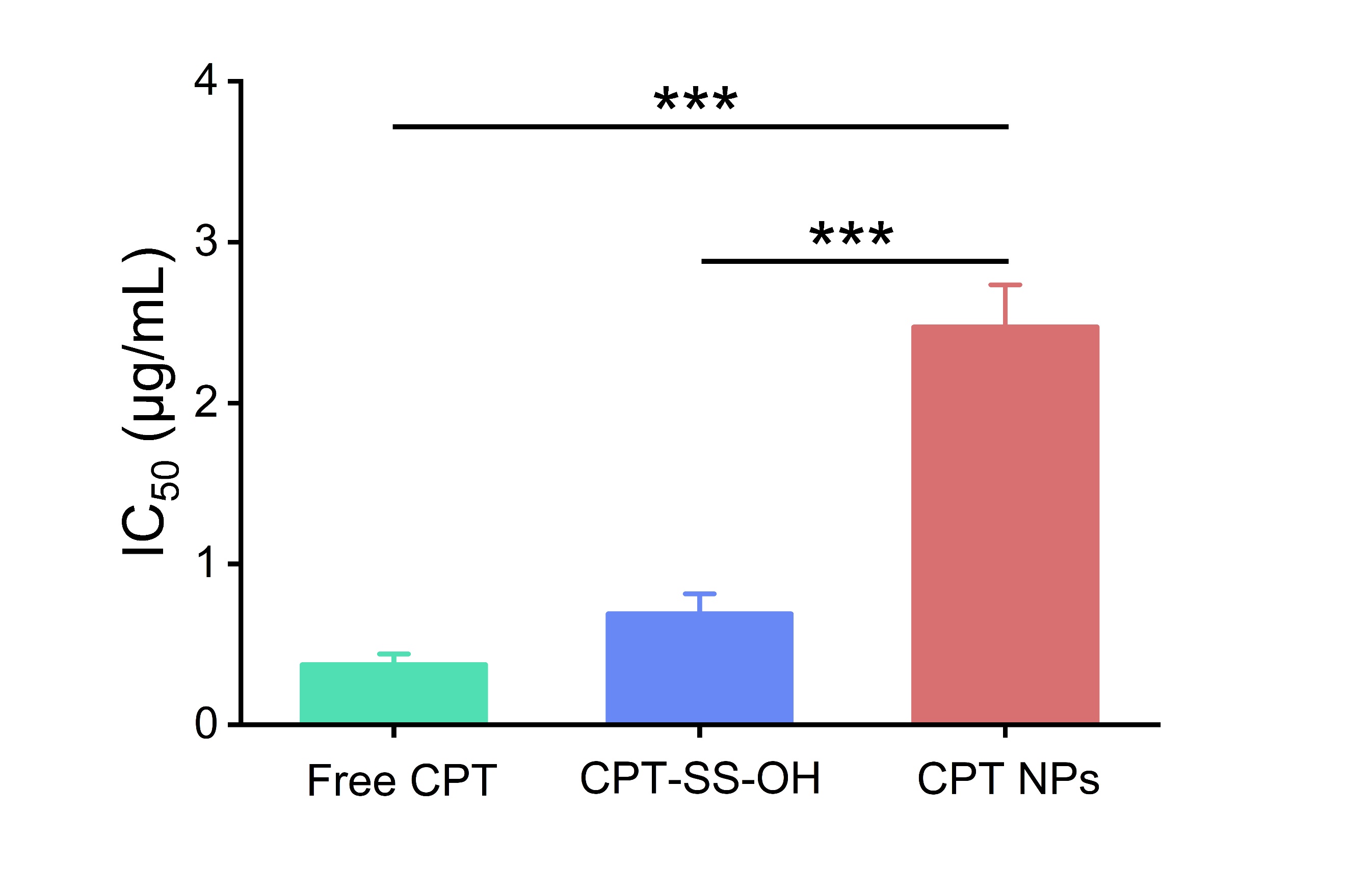


**Figure S6.** IC_50_ values of various drug formulations.

**Figure S7.** Synthetic route of PEG-CBA.


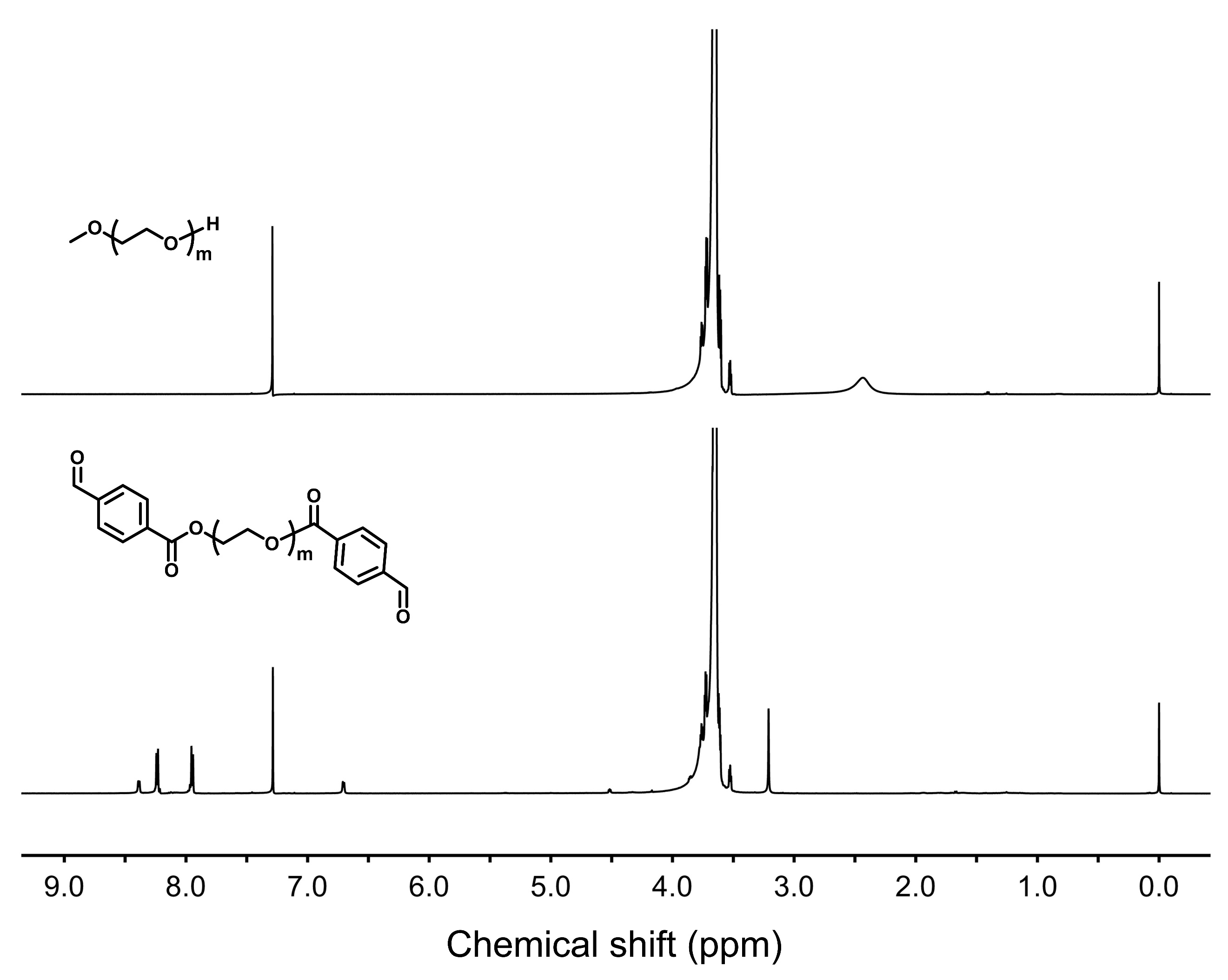


**Figure S8.** ^1^H NMR spectra of PEG and PEG-CBA.


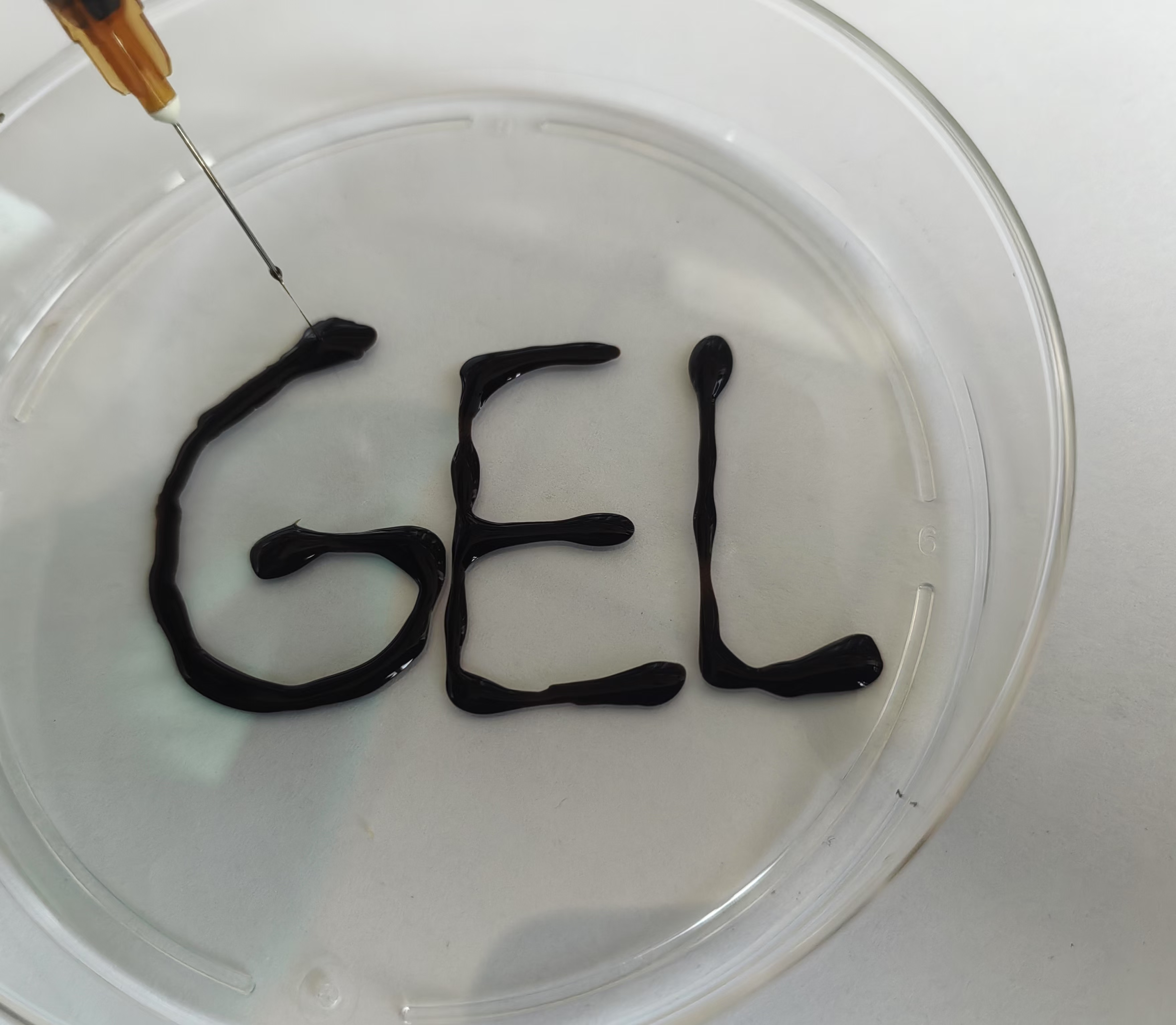


**Figure S9.** Injectability of CPT NPs gel.


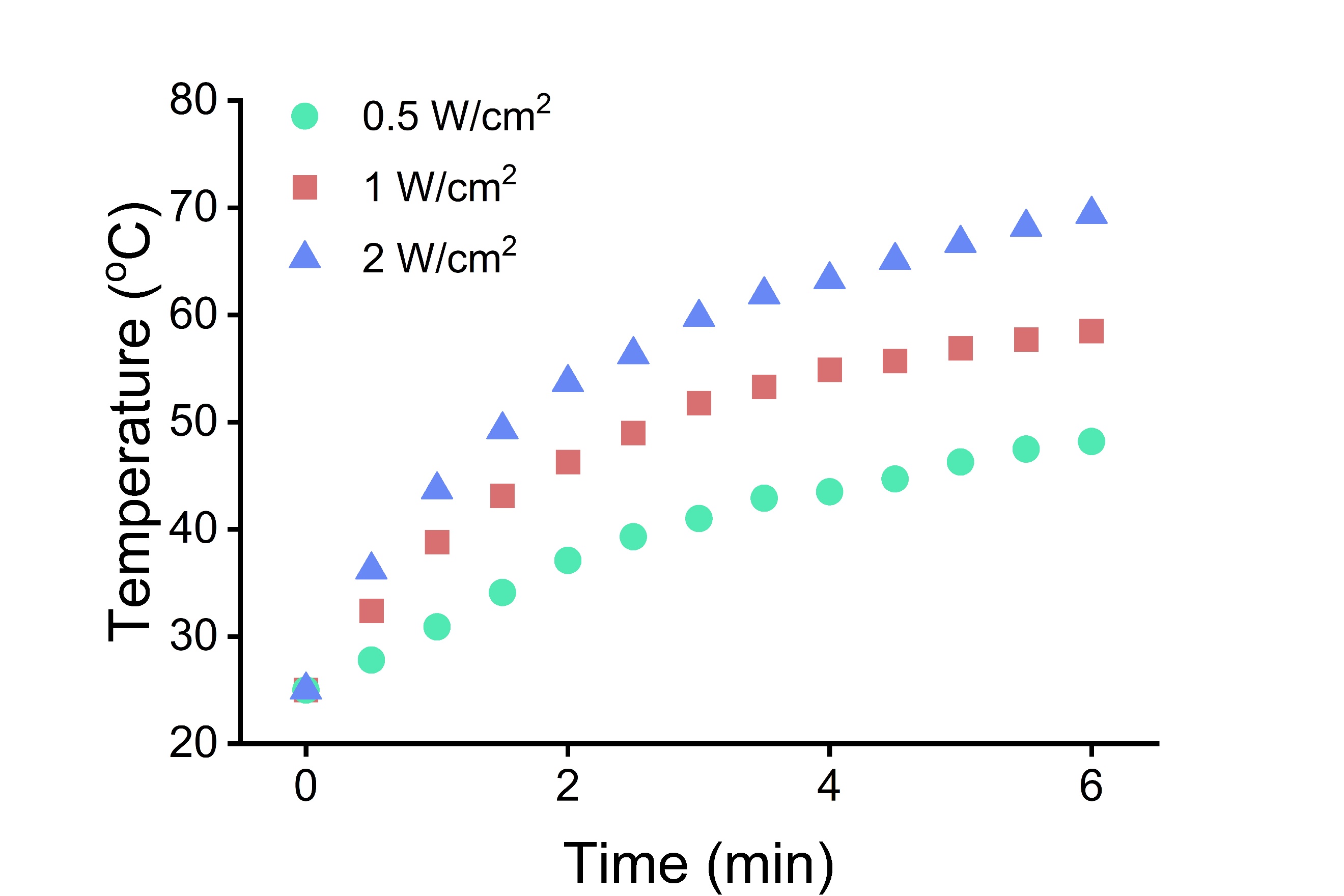


**Figure S10.** Temperature elevation profiles of CPT NPs gel under 808 nm laser irradiation at different power densities.


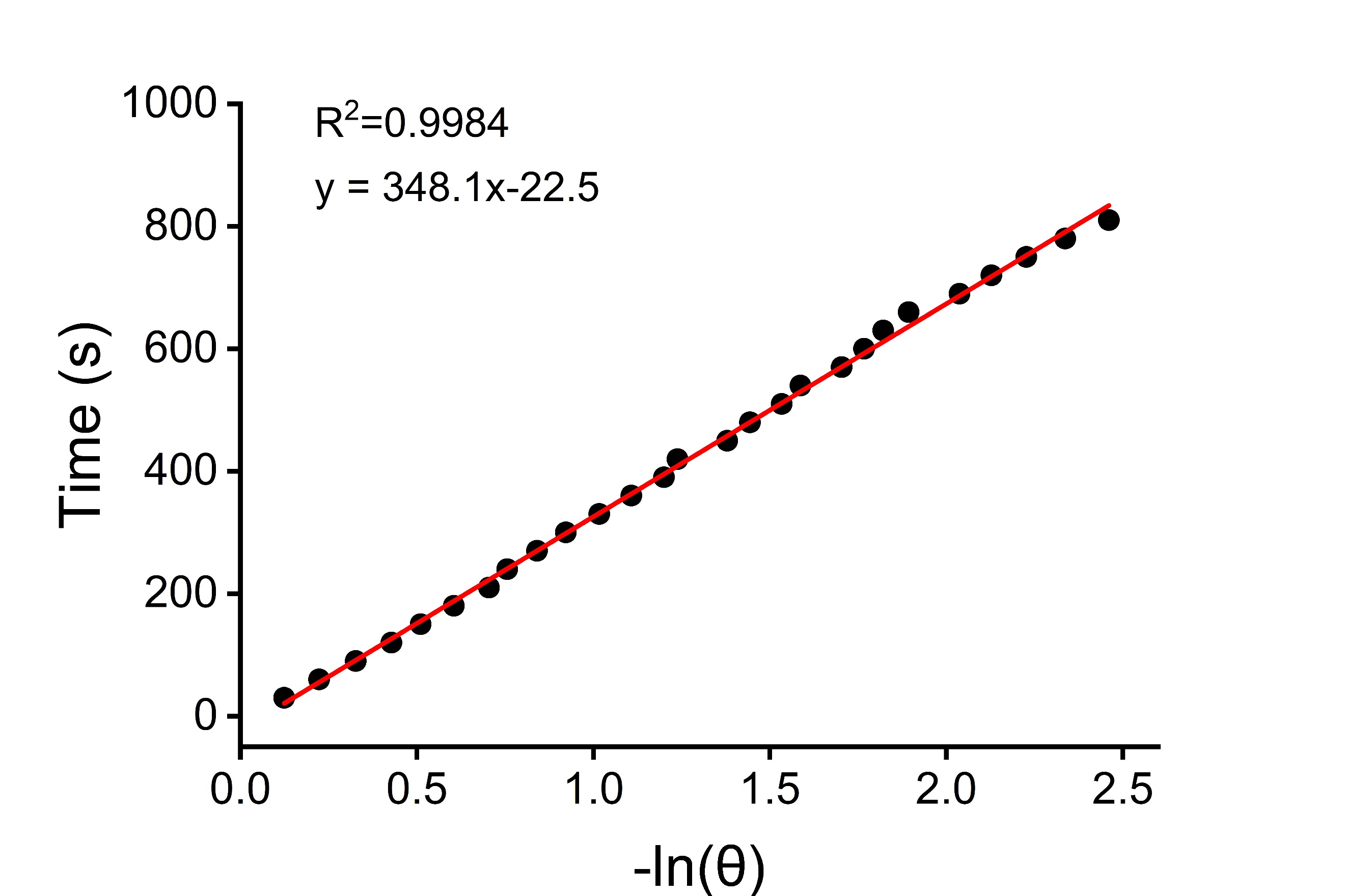


**Figure S11.** Linear relationship of time versus –lnθ derived from the first cooling curve.


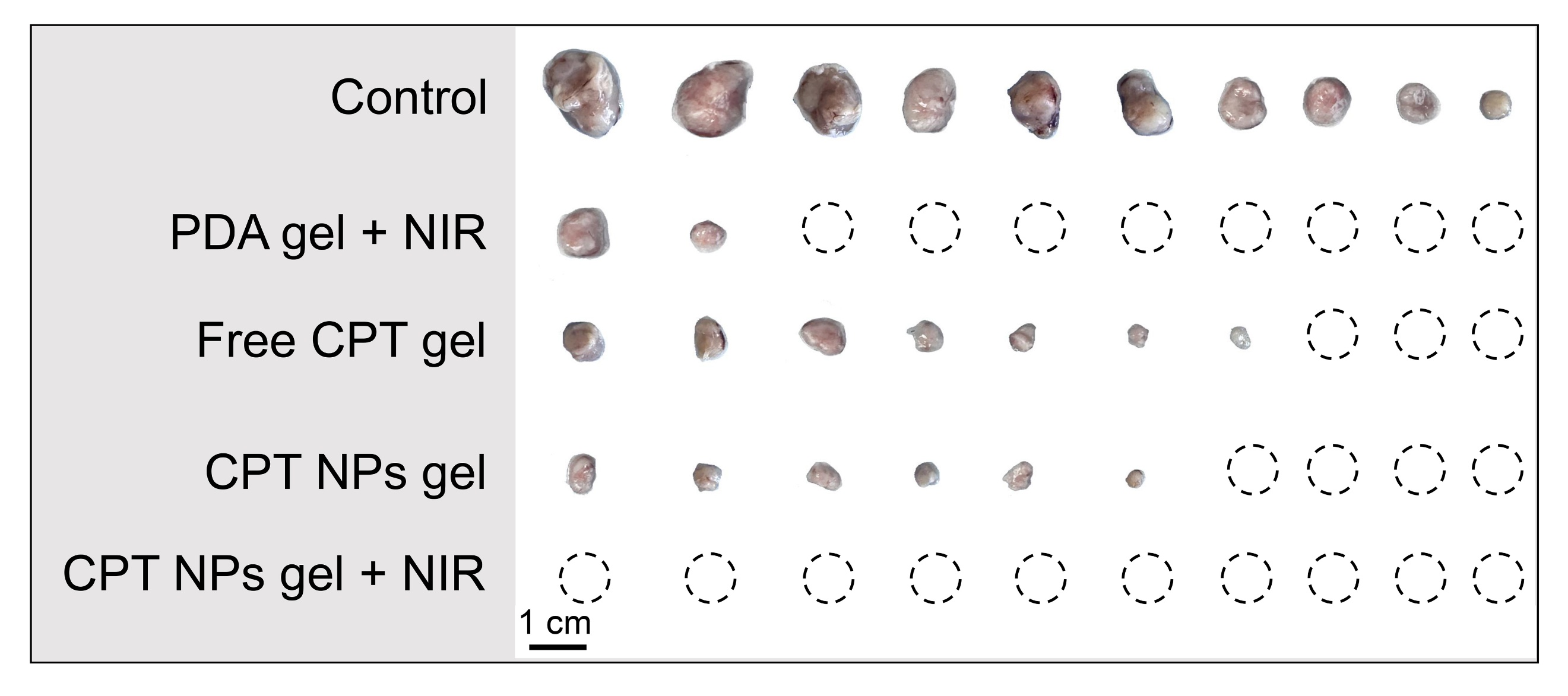


**Figure S12.** Tumor photos of each group on day 30.


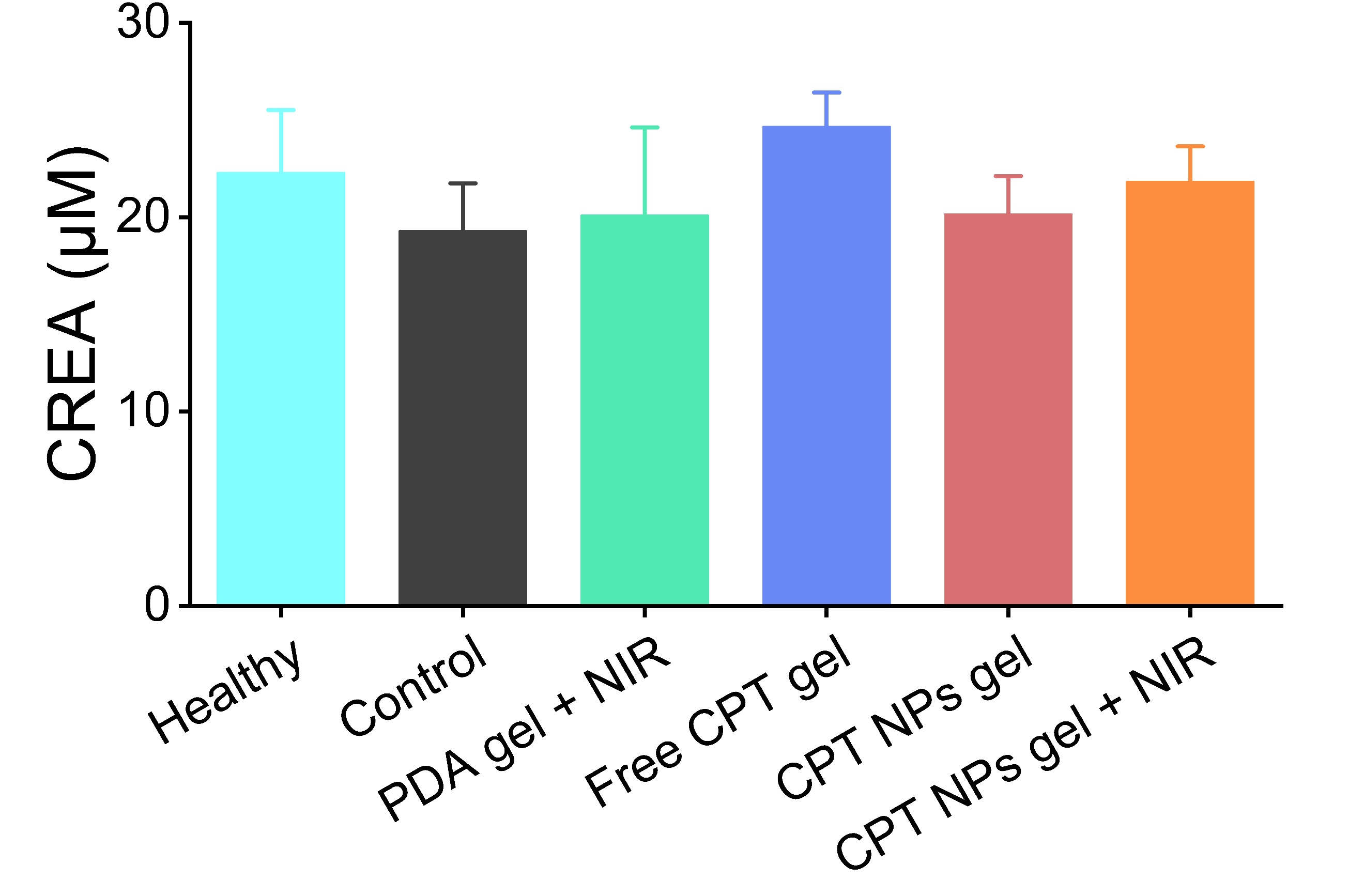


**Figure S13.** Level of CREA in healthy mice and post-treatment mice.


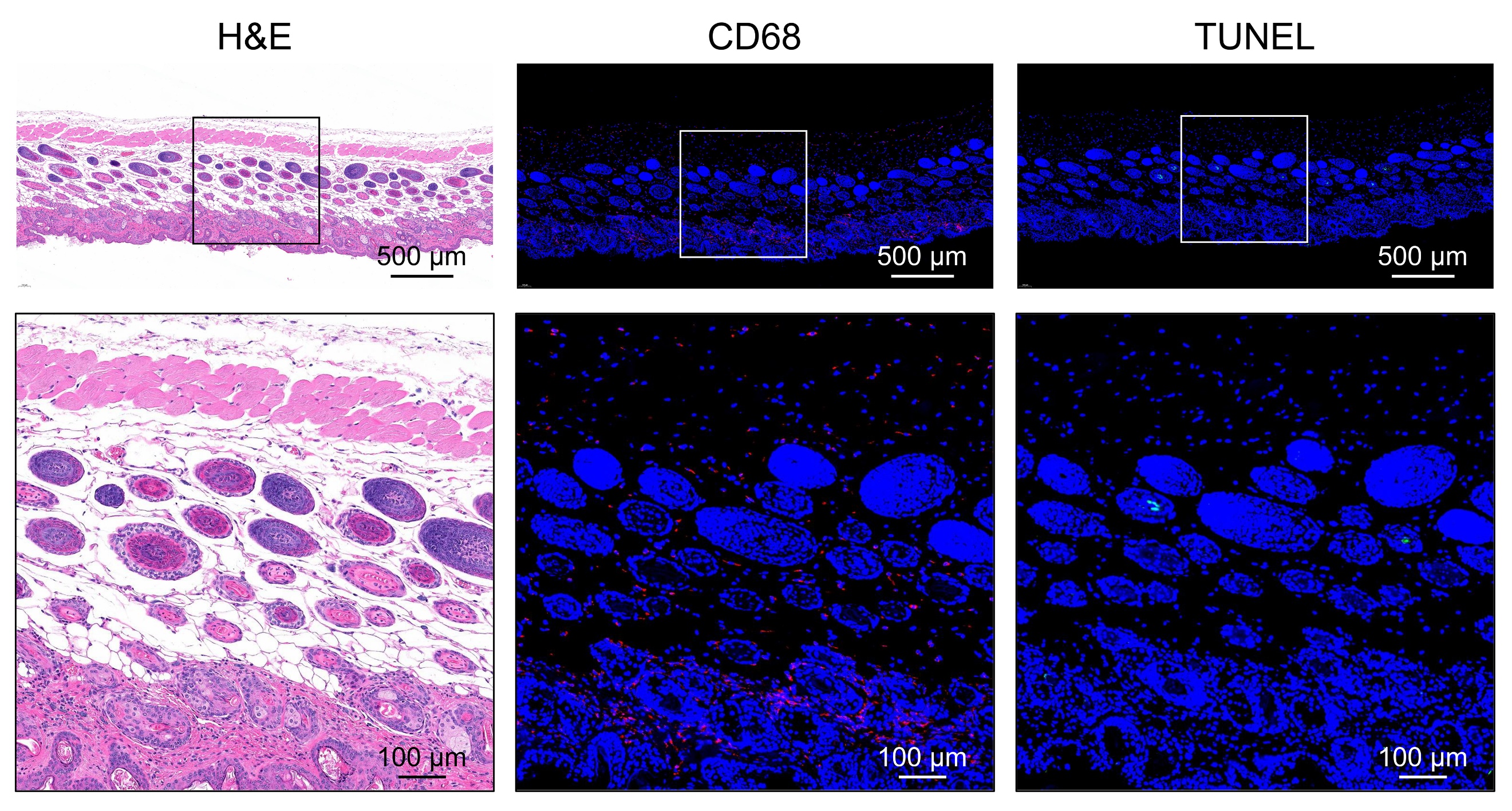


**Figure S14.** H&E staining and CD68 (red)/TUNEL (green) immunofluorescence images of skin tissues adjacent to the surgical resection site in the CPT NPs gel + NIR group. Cell nuclei in the immunofluorescence images were counterstained with DAPI (blue).


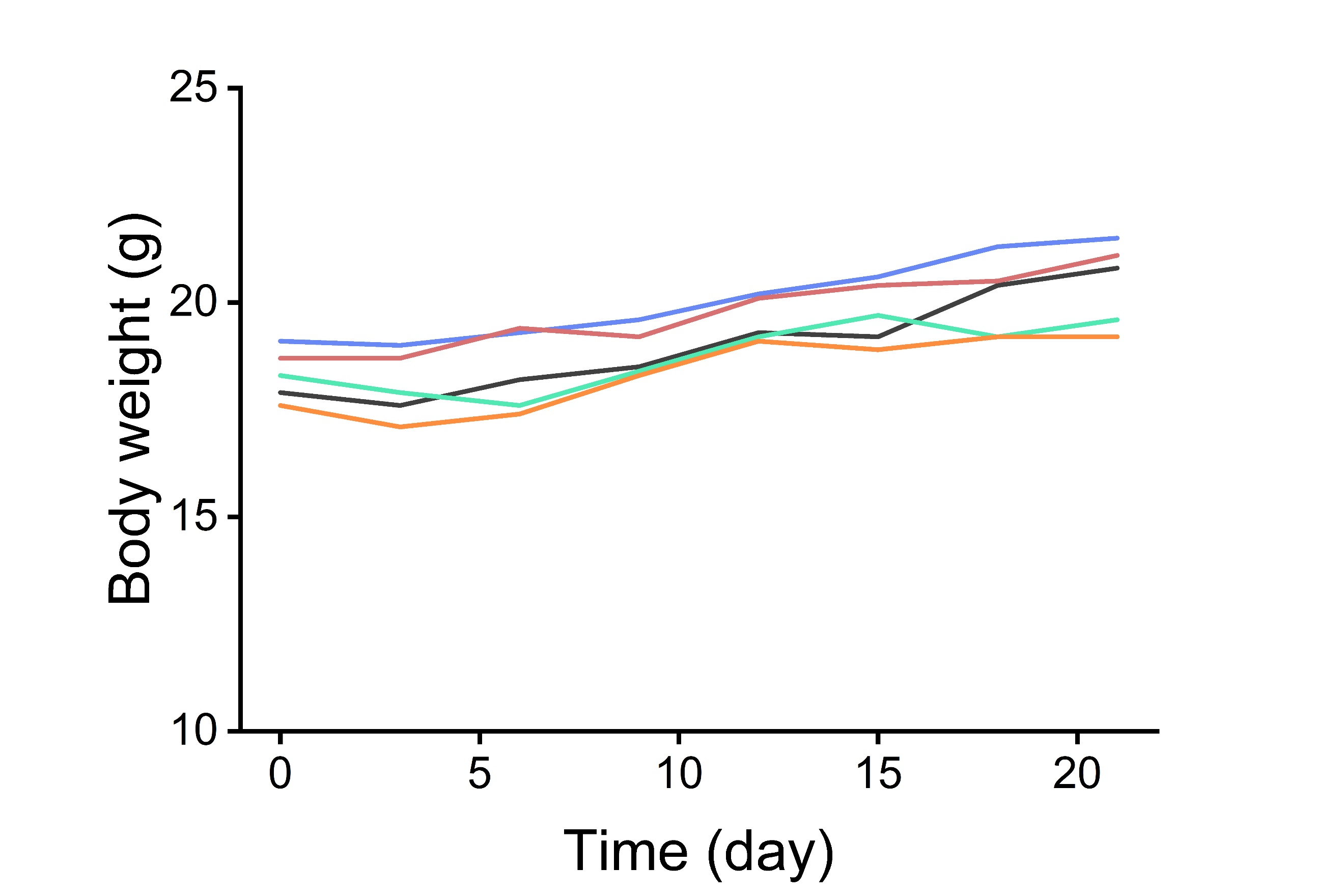


**Figure S15.** Body weight changes of healthy mice treated with CPT NPs gel + NIR.


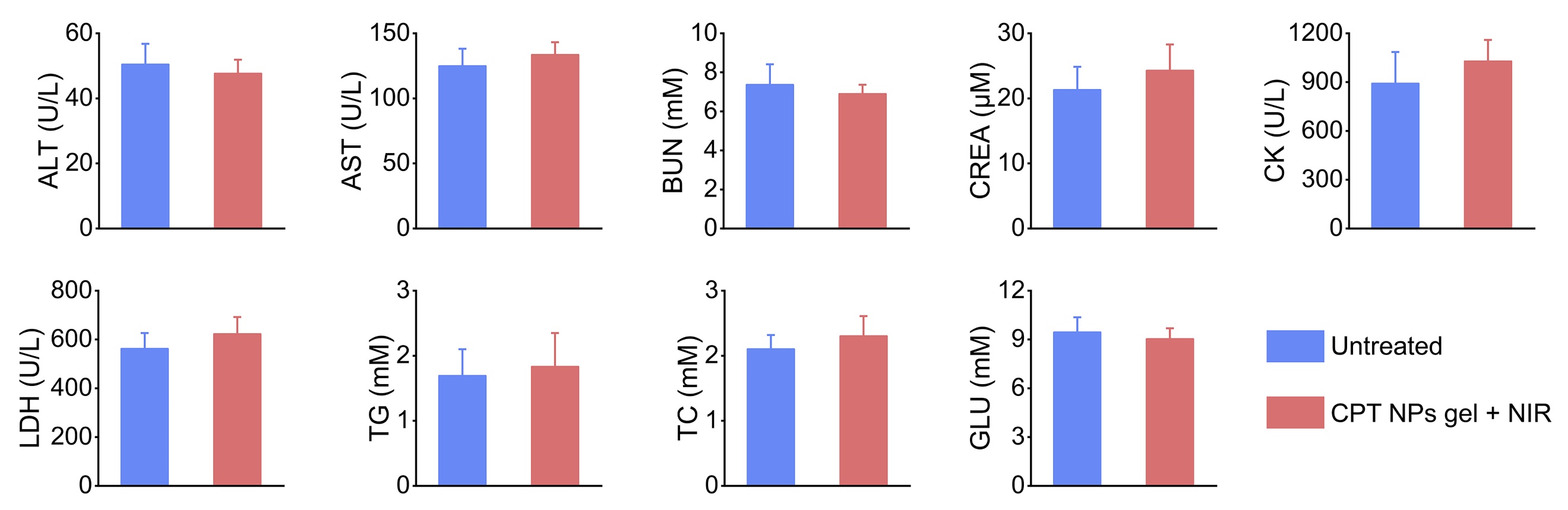


**Figure S16.** Serum levels of ALT, AST, BUN, CREA, CK, LDH, TG, TC, and GLU in healthy mice on day 21 after CPT NPs gel + NIR treatment.


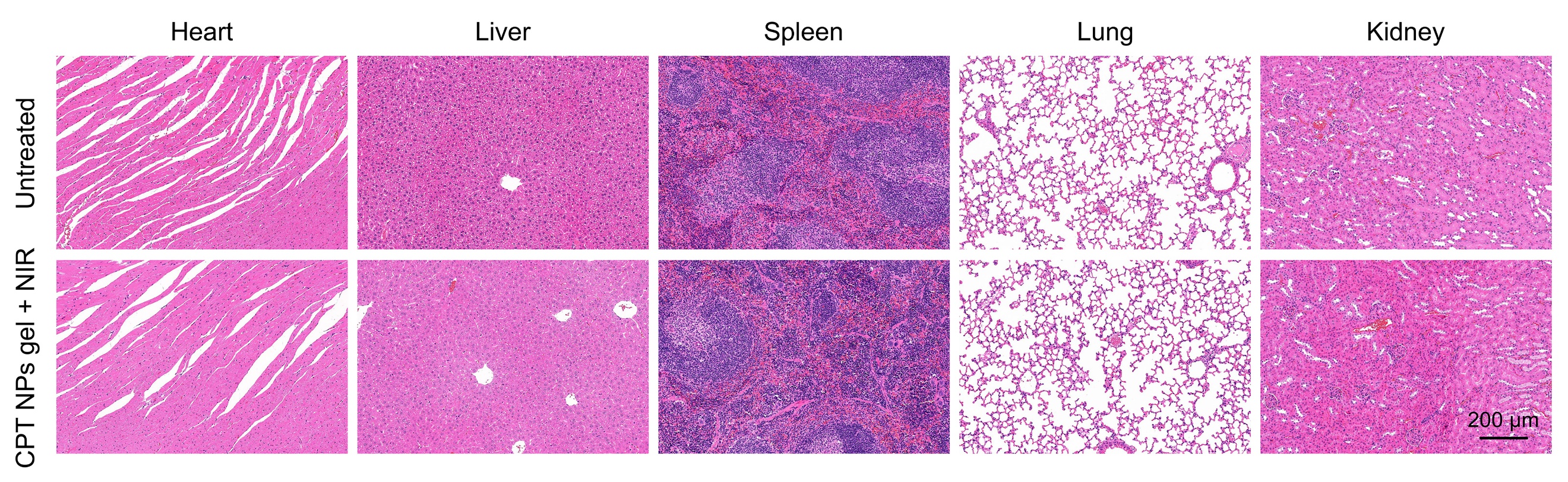


**Figure S17.** H&E analysis of the major organs from healthy mice on day 21 after CPT NPs gel + NIR treatment.


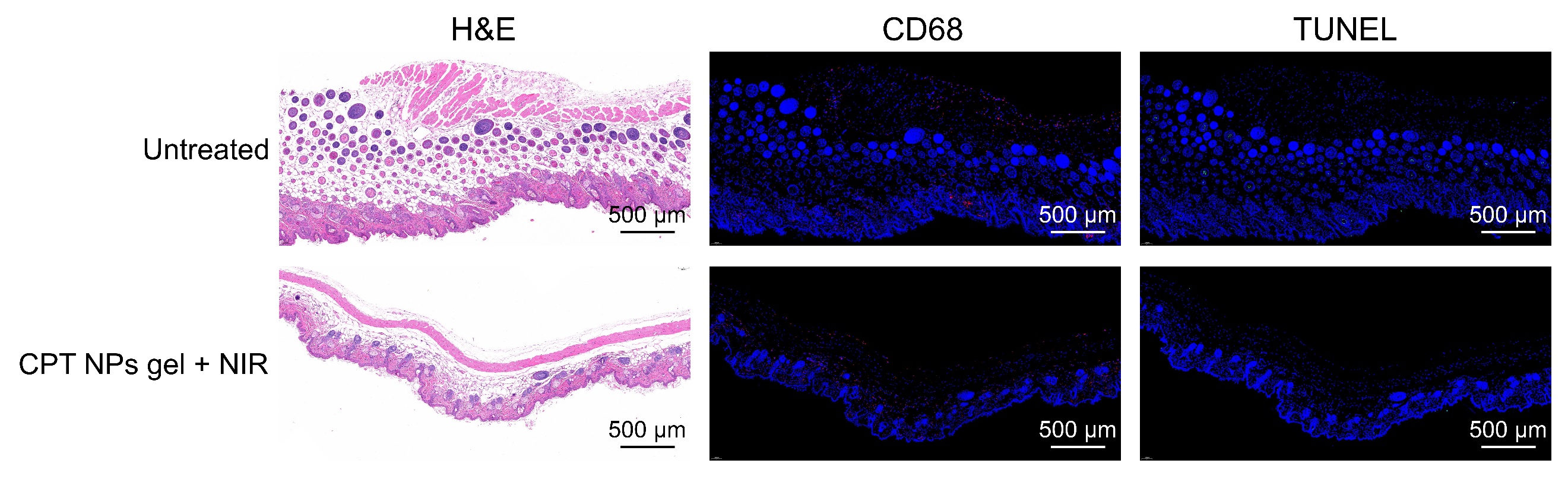


**Figure S18.** H&E staining and CD68 (red)/TUNEL (green) immunofluorescence images of skin tissues adjacent to the irradiation site in healthy mice.

## References

[1] Z. Guo, X. Liu, Z. Chen, J. Hu, L. Yang, New liquid crystal polycarbonate micelles for intracellular delivery of anticancer drugs, Colloid. Surfaces. B 178 (2019) 395–403.
